# Dynamic changes in transcriptome and cell wall composition underlying brassinosteroid-mediated lignification of switchgrass suspension cells

**DOI:** 10.1186/s13068-017-0954-2

**Published:** 2017-11-30

**Authors:** Xiaolan Rao, Hui Shen, Sivakumar Pattathil, Michael G. Hahn, Ivana Gelineo-Albersheim, Debra Mohnen, Yunqiao Pu, Arthur J. Ragauskas, Xin Chen, Fang Chen, Richard A. Dixon

**Affiliations:** 1 BioDiscovery Institute and Department of Biological Sciences, University of North Texas, Denton, TX USA; 2 BioEnergy Science Center (BESC), Oak Ridge National Laboratory (ORNL), Oak Ridge, TN USA; 3 Complex Carbohydrate Research Center, The University of Georgia, 315 Riverbend Rd, Athens, GA 30602 USA; 4 Department of Chemical and Biomolecular Engineering, University of Tennessee, Knoxville, TN USA; 5 Present Address: Marker-assisted Breeding and Traits, Chromatin Inc, Lubbock, TX 79404 USA; 6 Present Address: Center for Applied Mathematics, Tianjin University, Tianjin, 300072 China; 7 Present Address: Mascoma LLC (Lallemand Company), 67 Etna Road, Lebanon, NH 03766 USA

**Keywords:** Brassinosteroid, Cell wall, Comparative transcriptomics, Glycome profiling, Lignin, Suspension cell

## Abstract

**Background:**

Plant cell walls contribute the majority of plant biomass that can be used to produce transportation fuels. However, the complexity and variability in composition and structure of cell walls, particularly the presence of lignin, negatively impacts their deconstruction for bioenergy. Metabolic and genetic changes associated with secondary wall development in the biofuel crop switchgrass (*Panicum virgatum*) have yet to be reported.

**Results:**

Our previous studies have established a cell suspension system for switchgrass, in which cell wall lignification can be induced by application of brassinolide (BL). We have now collected cell wall composition and microarray-based transcriptome profiles for BL-induced and non-induced suspension cultures to provide an overview of the dynamic changes in transcriptional reprogramming during BL-induced cell wall modification. From this analysis, we have identified changes in candidate genes involved in cell wall precursor synthesis, cellulose, hemicellulose, and pectin formation and ester-linkage generation. We have also identified a large number of transcription factors with expression correlated with lignin biosynthesis genes, among which are candidates for control of syringyl (S) lignin accumulation.

**Conclusion:**

Together, this work provides an overview of the dynamic compositional changes during brassinosteroid-induced cell wall remodeling, and identifies candidate genes for future plant genetic engineering to overcome cell wall recalcitrance.

**Electronic supplementary material:**

The online version of this article (10.1186/s13068-017-0954-2) contains supplementary material, which is available to authorized users.

## Background

The plant cell wall has unique characteristics, with a dynamic architecture and composition that determines cell shape, supports plant mechanical structure during growth, and responds to developmental and environmental cues such as nutrient uptake and biotic/abiotic stress [1]. The cell wall contributes to the biomass of plants as the major carbon sink because plants assimilate much of the atmospheric carbon dioxide through photosynthesis into cell wall polymers including cellulose, hemicellulose, and lignin [2]. Although plant biomass is considered a promising source of reduced carbon for bioenergy, the conversion of lignocellulosic biomass into liquid transportation fuels is limited by the innate resistance of cell walls to microbial and enzymatic deconstruction [3], a phenomenon termed recalcitrance [4]. An understanding of the molecular mechanisms underlying the dynamic structure of plant cell walls in dedicated bioenergy crops is required for overcoming cell wall recalcitrance and enhancing biofuel production.

The grasses (family Poaceae) represent a major carbohydrate and protein resource for feeding humans and herbivores, as well as for biofuel production [5]. The family includes maize, Miscanthus, and switchgrass (*Panicum virgatum* L.), which have been selected as preferred feedstocks for bioenergy in the United States [5]. Grasses have distinct cell wall compositions compared with dicots [6]. Generally, the primary cell wall in grasses comprises assemblies of cellulose microfibrils embedded in a matrix of arabinoxylan with mixed-linkage glucans [7] and small amounts of pectin. Secondary cell walls in grasses exhibit a significant proportion of lignin as the major non-cellulosic component [7, 8], and this lignin generally possesses higher levels of syringyl (S) units and more esterified *p*-coumaric acid than dicot lignins [9, 10].

In spite of the interest in switchgrass as a bioenergy crop, few studies have been undertaken to elucidate cell wall structure and genes involved in cell wall biogenesis in this species. We previously generated a list of candidate genes involved in lignin biosynthesis in switchgrass by a combination of bioinformatic analysis and gene silencing technology [11], and baseline internode structure and cell wall composition have been determined in mature stems [12] and maturing tillers [13]. However, a comprehensive understanding of the genetic control of cell wall composition in switchgrass is absent, probably due to the complexity and variability of cell wall development *in planta*.

Compared with whole plants, suspension-cultured cells offer a simplified model system with a large population of relatively homogenous cells capable of rapid and uniform response to external stimuli [14], and have been used as effective model systems for investigating plant cell wall structure and composition since the 1970s [15, 16]. The brassinosteroids (BRs) are a group of naturally occurring plant hormones that function in cell elongation and differentiation as well as cell wall formation [17]. Exogenous supplementation of brassinolide (BL) can induce tracheary element formation and the deposition of cellulose and lignin during secondary wall formation in suspension-cultured Arabidopsis, Zinnia *(Zinnia elegans*), and banana embryonic cells [18–21].

**Fig. 1:**
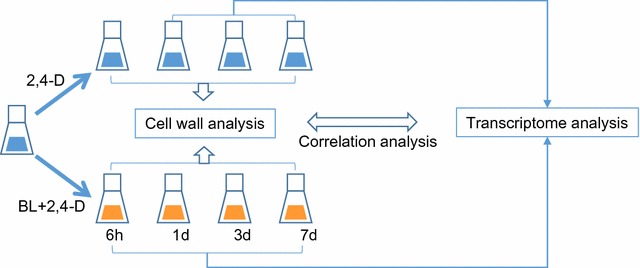
Experimental design. Switchgrass cell suspension cultures were transferred to fresh medium containing 9 μM 2,4-D (controls, blue), or to medium with 0.9 μM 2,4-D supplemented with 0.2 μM BL (induced, orange), and harvested at the times shown. The changes in cell wall composition of induced and non-induced samples were analyzed by biochemical and immunochemical approaches at all the times shown. Microarray analysis was performed on induced samples harvested at 0, 6 h, 1, 3, and 7 days after transfer, and on non-induced samples 1 and 7 days after transfer. Comparative transcriptomics and correlation with changes in cell wall composition were applied to identify putative cell wall-related genes

Previously, we established a suspension culture system with cells generated from callus derived from immature inflorescences of switchgrass variety Alamo [11, 22]. As in a *Pinus taeda* suspension cell system [23], only primary cell walls form in switchgrass suspension cultures grown in medium containing high concentrations (9 μM) of 2,4-dichlorophenoxyacetic acid (2,4-D) [11]. Supplementation with BL (0.2 μM) along with a simultaneous reduction in the concentration of 2,4-D (to 0.9 μM) induces formation of secondary cell wall-associated lignification by 7 days of treatment [11]. Phloroglucinol-HCl staining showed that BL-induced lignin is located within the cells, and no lignin is released into the culture medium [11]. We have now applied biochemical, immunochemical, and NMR analyses, along with global transcript profiling, to determine the changes in cell wall composition and associated gene expression in both BL-induced- and non-induced switchgrass suspension cultures, which are associated with development of primary and secondary cell walls, or only primary cell walls, respectively (Fig. 1). The combined information provides insights into the genetic control of the dynamic changes in cell wall composition associated with the switch from primary to secondary wall formation.

## Results and discussion

### Changes in cell wall glycosyl residues

**Fig. 2:**
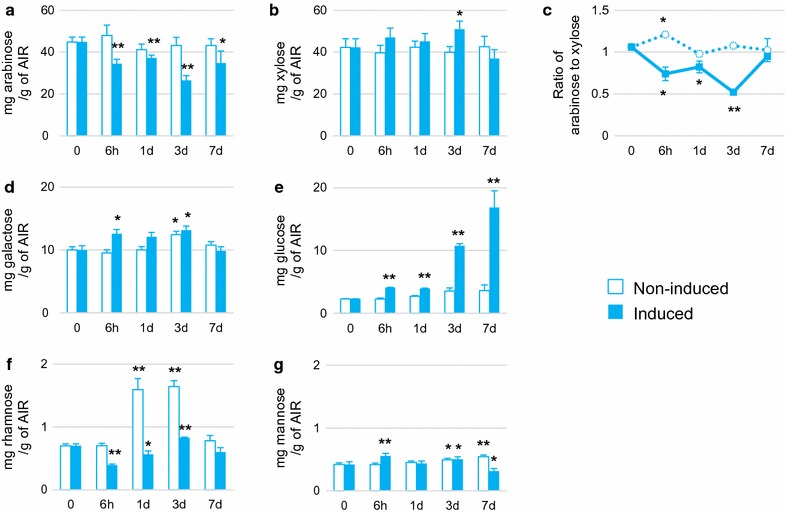
Monosaccharide composition of cell wall residues isolated from non-induced and BL-induced switchgrass cultures at the times indicated. Panels **a**, **b**, **d**, **e, f**, and **g** indicate arabinose, xylose, galactose, glucose, rhamnose, and mannose contents, respectively. **c** Indicates the ratio of arabinose to xylose. Non-induced and induced samples are presented as open bars/dash line and closed bars/solid line, respectively. All data are mean ± SE (*n* = 3). The significant differences from 0 h control were determined by the Student’s *t* test and represented by a single asterisk (*p* < 0.05) or double asterisk (*p* < 0.01). The significant differences by the Student’s *t* test between induced and non-induced samples at each time point are provided in Additional file 11: Table S8

The sandy type of switchgrass cell culture that attains high cell density [22] was selected for this work because of its rapid and uniform growth [11]. No obvious changes in cell morphology were observed between BL-induced and non-induced sandy suspension cultures [11]. To assess the extent to which cell wall composition is altered during BL-induced lignification, we first analyzed the monosaccharide composition of cell walls from induced and non-induced suspension cultures as a function of time after BL addition (Fig. 2). The major monosaccharide components of the alcohol-insoluble residue (AIR) from the cell walls were quantified using GC–MS after hydrolysis.

The monosaccharide composition of the switchgrass cell wall preparations at the time of transfer (initial stage, 0 h) was similar to that previously reported for cell walls of suspension cultures of five other grasses; arabinose and xylose were the most abundant components and the ratio of arabinose to xylose was approximately equal to 1 (Additional file 1: Figure S1). Arabinoxylan (AX) is considered to be the major hemicellulosic structural component in which cellulose is embedded in grass cell walls [1, 15]. The content of arabinose was reduced from 6 h to 3 days following application of BL (Fig. 2a), whereas the content of xylose showed the opposite trend (Fig. 2b), leading to a significant decrease in the ratio of arabinose to xylose during this period (Fig. 2c). In contrast, levels of arabinose and xylose remained relatively constant in non-induced suspension cultures except for a slight increase in the ratio of arabinose to xylose at 6 h. AX consists of a backbone of β-1,4-linked xylose residues on which the O3 position is frequently substituted by arabinose side chains [24]. The ratio of arabinose to xylose therefore reflects the degree of linearity or branching of this hemicellulose [24]. Our data indicate that increased levels of linear xylan regions may occur in switchgrass suspension cells between 6 h and 3 days post-treatment with BL.

Other glycosyl residues, such as galactose (Fig. 2d) and mannose (Fig. 2g), are less abundant in the hemicellulosic fraction of grass cell walls [7], but changes in these sugars, likely in galactoarabinoxylans and galactomannans, also occur during induced lignification (Fig. 2d, g).

A large increase in the level of cell wall glucose was observed in BL-induced suspension cultures compared to non-induced cultures (Fig. 2e). Use of β-glucan antibodies (see section on glycome profiling) suggests that a significant increase in non-cellulosic polymers contributes to the increase in glucose content in BL-induced cells. In addition, we observed a significant increase in cell wall rhamnose in non-induced cultures at days 1 and 3, with no corresponding increase in induced cultures at the same times (Fig. 2f). Rhamnose is a component of the rhamnogalacturonan (RG)-II and RG-I pectic polysaccharides of the cell wall [25]. These results suggest that deposition of pectic polysaccharides is halted in the cell walls of BL-induced cells associated with the induction of lignification.

### Changes in cell wall extractability

**Fig. 3:**
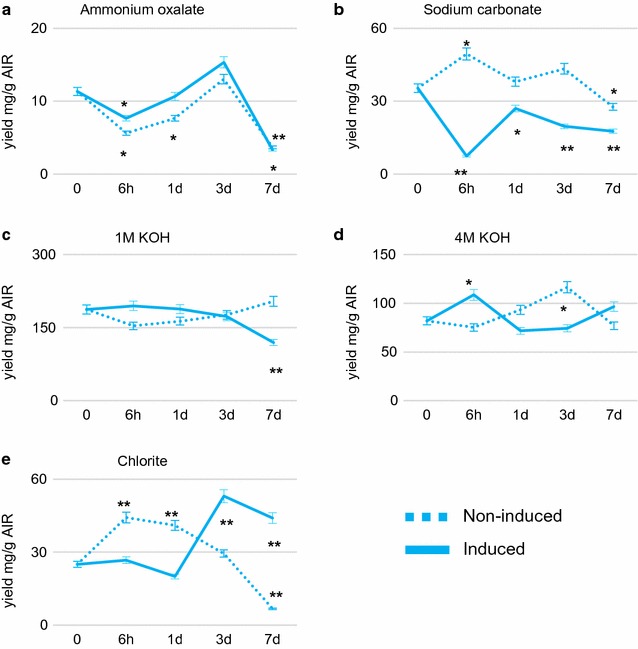
Amounts of polysaccharide sequentially extracted from cell wall residues of non-induced and BL-induced cultures using increasingly harsh solvents at the times shown. **a**–**e** indicate total carbohydrate yield in the ammonium oxalate, sodium carbonate 1 M KOH, 4 M KOH, and chlorite fractions, respectively. Non-induced and induced samples are present as dash line and solid line, respectively. All data are mean ± SE (*n* = 3). The significant differences from the 0-h control were determined by the Student’s *t* test and are represented by a single asterisk (*p* < 0.05) or double asterisk (*p* < 0.01). The significant differences by the Student’s *t* test between induced and non-induced samples at each time point are provided in Additional file 11: Table S8

As a prelude to glycome profiling (see below), the AIR preparations analyzed in Fig. 2 were extracted sequentially with increasingly harsh reagents (ammonium oxalate, sodium carbonate, sodium chlorite, 1 M KOH, and 4 M KOH) to release the most soluble to the most tightly bound cell wall components [26], and the total sugar content was determined in each extract (Fig. 3).

Ammonium oxalate and sodium carbonate extracts are usually enriched in pectins and arabinogalactans [27]. Ammonium oxalate releases pectic polymers that are loosely bound to the wall by ionic interactions, whereas sodium carbonate extracts polymers by hydrolysis of base-labile bonds (e.g., sensitive esters such as methylesters) or as a consequence of increased negativity of deprotonated chemical (e.g., carboxyl) groups and/or due to chelation of remaining cations (e.g., calcium) [28]. Ammonium oxalate extracted similar amounts of material from induced and non-induced cell cultures during the time course, with a large reduction in extractable material at day 7 in both cases (Fig. 3a). In contrast, sodium carbonate released less material from BL-induced cell cultures compared to non-induced cell cultures, especially at the 6-h and 3-day time points (Fig. 3b). This suggests that BL treatment does not affect the content of loosely bound pectins and arabinogalactans, but rather causes a reduction in a fraction of these molecules that are more tightly held in the wall by base-sensitive linkages and/or tight ionic binding.

The material extracted by 1 M and 4 M KOH is typically enriched in hemicelluloses, but usually also contains tightly bound pectins, arabinogalactans, and phenolics. More material was released by 1 M KOH than by 4 M KOH in both induced and non-induced samples (Fig. 3c, d). Decreasing amounts of 1 M KOH-extractable material were released from the AIR from BL-induced cells during the time course, whereas the opposite was the case for the non-induced samples (Fig. 3c, d). In the 4 M KOH fractions from induced samples, a peak of extractable material was seen at 6 h post-induction, whereas the peak was delayed to 3 days in the non-induced cells (Fig. 3d). Hemicellulosic components with a higher degree of hydrogen bonding require more concentrated alkali for extraction [29]. The data therefore suggest that, after BL treatment, the content of hemicellulose with lower degree of hydrogen bonding may decrease and the content of hemicellulose with a higher degree of hydrogen bonding may reach a peak around 6 h post-treatment.

Finally, the amount of material extracted by sodium chlorite was very different from walls of induced and non-induced cell cultures (Fig. 3e). This material consists of phenolic-associated wall polysaccharides [30]. In non-induced cultures, there was an increase in material released at 6 h and a reduction thereafter. In contrast, chlorite-extractable material decreased slightly up to 1 day and then increased in induced samples (Fig. 3e). These changes may be associated with the changes in lignification observed in the cultures (see below).

### Glycome profiling reveals BL-induced changes in cell wall composition

To gain a more complete picture of cell wall compositional changes, cell wall fractions extracted with the reagents above were subjected to glycome profiling, which detects specific glycan epitopes using monoclonal antibodies [26] (Additional file 1: Figure S2). The glycan-directed antibodies used account for the majority of non-cellulosic glycan components of plant cell walls [7]. Using this approach, we observed a series of differences in the extractability of glycan components from walls of both non-induced and induced cells during the time course.

**Fig. 4:**
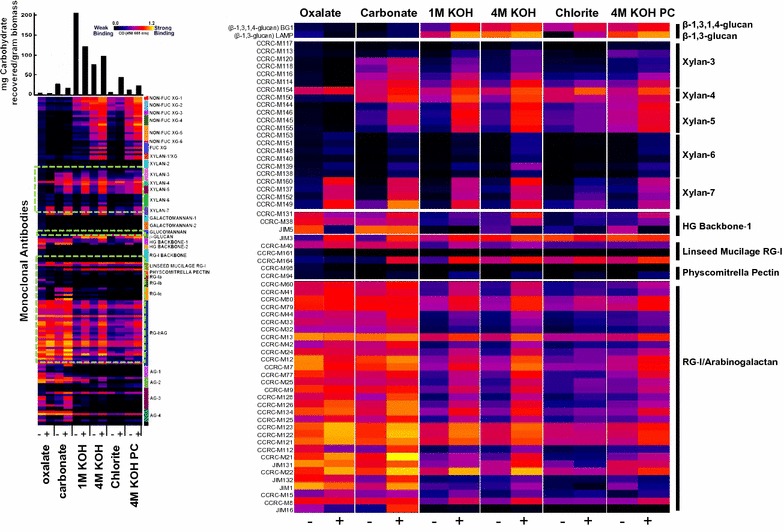
Glycome profiling heat maps showing the relative abundance of cell wall glycan epitopes released sequentially from the AIR fraction of 7-day BL-induced and non-induced suspension cells. Plus and minus symbols indicate BL-induced and non-induced samples, respectively. The cell walls were sequentially extracted with ammonium oxalate, sodium carbonate, 1 M and 4 M KOH, chlorite, and 4 M KOH post-chlorite as indicated. The solubilized extracts were then analyzed by ELISA using cell wall glycan-directed monoclonal antibodies. The results presented on the heatmaps are the average values of three independent replicates. Right panel shows a magnified view of the hatched areas on the left panel

The most striking differences were observed with the Xylan-7 antibodies, which showed increased binding in the induced cultures at 7 days in all extracts (Fig. 4 and Additional file 1: Figure S2). Other xylan epitopes recognized by the Xylan-4 and -5 clades of antibodies (which are selective for side chains on xylans [31] also showed increases in induced cultures at 3 and 7 days in the 1 M KOH, 4 M KOH, and 4 M KOH PC extracts. These increases in xylan epitopes are consistent with the observed induction of secondary wall formation in the induced cultures. Other more subtle increases were also observed in induced cultures for xyloglucan epitopes at 7 days in the 4 M KOH and 4 M KOH PC extracts (Fig. 4 and Additional file 1: Figure S2) and for pectic arabinogalactan epitopes (recognized by the RG-I/AG antibodies) at 7 days in all extracts (Fig. 4). An increase in the latter epitopes was also observed in induced cultures at 3 days in the 1 M KOH and 4 M KOH extracts (Additional file 1: Figure S2).

A closer inspection of the antibody reactive epitopes expressed in induced versus non-induced cell wall material extracted from cells at 7 days revealed a subtle increase in homogalacturonan and mixed-linkage β-glucan epitopes in induced compared to non-induced cultures, particularly in the 1 M KOH and 4 M KOH fractions (Fig. 4). We propose that these changes upon BL-induction are associated with secondary cell wall development. The glycome profiles suggest that our previous observation of a BL-induced increase in glucose in cell wall preparations may reflect an increase in non-cellulosic mixed-linkage (1 → 3, 1 → 4) β-glucans and possibly (1 → 3) β-glucans (calluses).

### Lignin and hydroxycinnamate accumulation

Previously, we had shown that the level of lignin continuously increased in switchgrass cell suspension cultures after 2 days following BL treatment, while only trace amounts of lignin accumulated in non-induced suspension cultures during the 7-day time period [11]. To further elucidate the nature of phenylpropanoid-derived compounds induced in BL-supplied suspension cells, HPLC, GC–MS, and 2D heteronuclear single-quantum coherence (HSQC) Nuclear Magnetic Resonance (NMR) spectroscopy were applied to analyze and quantify phenolic groups in lignins and esterified to cell wall components.

Wall-bound phenolic acids are embedded into cell walls through their chemical linkage with polysaccharides and polymeric lignin [32, 33]. *p*-Coumaric acid (p-CA) is preferentially ester-linked to lignin rather than to hemicellulose, whereas ferulic acid (FA) is exclusively ester-linked to glucuronoarabinoxylan in grass cell walls [32, 33]. The amount of p-CA reflects the degree of lignification in grasses and a high *p*-CA/FA ratio is considered to be highly correlated with cell wall recalcitrance [34]. In non-induced cells, FA was the most abundant wall-bound phenolic acid and essentially no *p*-CA was detected (Additional file 1: Figures S3A, B). In induced cells, the *p*-CA content increased while the wall-bound FA content decreased during the time course (Additional file 1: Figures S3A and B), leading to a large increase of *p*-CA/FA ratio (Additional file 1: Figures S3C). The increase in ester-linked *p*-CA and *p*-CA/FA ratio parallels the accumulation of lignin content in induced suspension cultures.

**Fig. 5:**
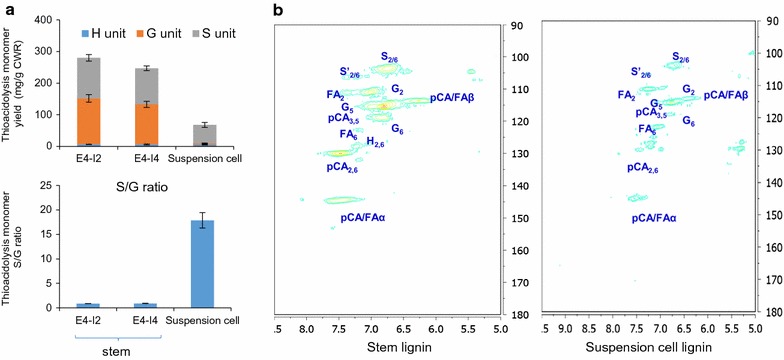
A comparison of lignin quantity and quality in switchgrass stem and BL-induced suspension culture. **a** Lignin quantity and composition as determined by thioacidolysis yield (upper panel), and S/G ratio (lower panel). **b** HSQC-NMR analysis of the aromatic region. Stem lignin was from plants at the E4–I2 and E4–I4 developmental stages for thioacidolysis analysis, and a mixture of E4–I2 and E4–I4 stages for NMR analysis. Cell cultures were 7 days post-treatment with BL

Predominantly S lignin monomers, with only trace amounts of G lignin, were detected in induced suspension cells using thioacidolysis followed by GC–MS (Fig. 5a). The lignin isolated from the induced suspension cells had a much higher S:G ratio than that isolated from switchgrass stems (Fig. 5a), though the lignin from the two sources shares a similar degree of polymerization [11, 74]. Because thioacidolysis preferentially analyzes β-*O*-4-linked lignin units, the overall enrichment in S lignin and deficiency in G lignin in induced suspension cells was further confirmed using 2D ^13^C–^1^H HSQC NMR analysis (Fig. 5b). Compared with that in switchgrass stem samples, the aromatic region of the 2D ^13^C–^1^H HSQC correlation NMR spectrum shows that the majority of lignin induced in switchgrass suspension cells is made of S units and contains a considerable amount of *p*-courmarate and ferulate, whereas G units are rare (Fig. 5b).

### Functional classification of differentially expressed genes during BL-induced cell wall modification

To obtain a genome-wide understanding of the transcriptional reprogramming associated with BL-induced changes in cell wall composition, microarray analyses were conducted on RNA extracted from cells harvested on 0, 6 h, 1, 3, and 7 days for induced suspension cell cultures, as well as 1 and 7 days for non-induced cultures [11]. Low expression genes were filtered from the microarray dataset and 58,079 probes remained for subsequent analysis. Principal component analysis (PCA) was performed to visually summarize the features of the gene expression profiles. The details of data pre-processing and principal component analysis (PCA) are described in Additional file 2: Methods.

**Fig. 6:**
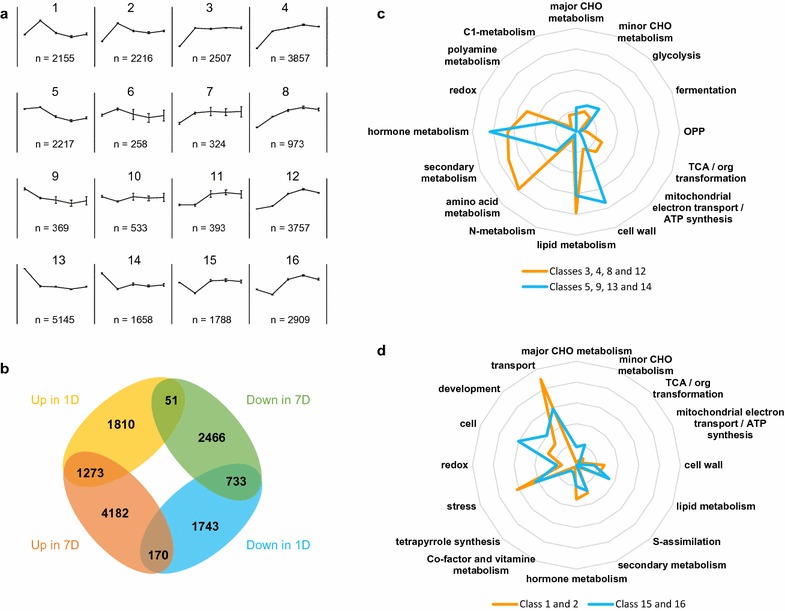
Cluster analysis of gene expression in BL-induced cultures and analysis of differentially expressed genes. **a** Cluster analysis of genes with at least twofold change in expression in induced cells using SOM method (4 × 4 matrix). **b** Numbers of differentially expressed genes between induced and non-induced cells on 1 and 7 days. **c** and **d** show relative numbers of gene functional groups in selected expression clusters

To optimally classify genes according to their expression pattern, we applied a self-organizing map (SOM) method to cluster gene sets with a 4 × 4 matrix (Fig. 6a) and defined sixteen expression groups of filtered genes during the 7-day time course in induced suspension cultures (Fig. 6a). Generally speaking, Clusters 3, 4, 8, and 12 represent genes for which transcript levels were gradually elevated after BL treatment, whereas the expression levels of genes in Clusters 5, 9, 13, and 14 were significantly decreased over the same period. Clusters 1 and 2 represent genes that responded rapidly to BL with transcript levels up-regulated at 6 h and restored after that time, whereas genes in Clusters 15 and 16 were expressed in the opposite way.

We compared the biological functions of differentially expressed genes between clusters using the Mapman [35] functional catalog database (Additional file 3: Table S1). We first compared the functional categories of transcripts in Clusters 3, 4, 8, and 12, and Clusters 5, 9, 13, and 14 (Fig. 6c) that consist of steadily up-regulated and steadily down-regulated genes following BL treatment, respectively. Eight thousand six hundred and ninety-nine out of the total of 11,094 genes in Clusters 3, 4, 8, and 12, and 7108 of the total of 9389 genes in Clusters 5, 9, 13, and 14 could be annotated into 35 main catalogs of Mapman based on the corresponding Arabidopsis orthologs. Among them, genes involved in the oxidative pentose phosphate (OPP) pathway, TCA cycle, mitochondrial electron transport/ATP synthesis, amino acid metabolism, nucleotide metabolism, and secondary metabolism were preferentially enriched in Clusters 3, 4, 8, and 12, whereas genes involved in glycolysis, fermentation and cell wall metabolism (including cellulose and hemicellulose synthesis) were significantly enriched in Clusters 5, 9, 13, and 14 (Fig. 6d and Additional file 3: Table S1). Taken together, these data are consistent with a gradual reduction in conversion of sucrose (the carbon source in the cultures) into complex carbohydrates (such as cellulose and hemicellulose) stored in the cell wall and an increased conversion of sucrose into amino acid, nucleotide, and secondary metabolism (with reductive power supplied by the OPP), through the intermediates of the TCA cycle following exposure to BL.

We also compared the functional categories of transcripts in Clusters 1 and 2, and Clusters 15 and 16 (Fig. 6d), which represent genes that were rapidly up-regulated or down-regulated in response to BL treatment, respectively. A total of 4371 and 4697 genes were grouped into Clusters 1 and 2 and Clusters 15 and 16, respectively, and 3420 and 3526 genes had orthologs in Arabidopsis annotated in the Mapman database, respectively. More genes involved in major and minor CHO metabolism were preferentially enriched in Clusters 15 and 16 compared with Clusters 1 and 2 (Fig. 6d and Additional file 4: Table S2). Further analysis in sub-categories indicates that the expression of 28 genes involved in sucrose and starch synthesis was rapidly reduced by 6 h and then recovered in response to BL treatment (Additional file 4: Table S2). Genes predicted to be involved in transport and cell division/growth were enriched in Clusters 1 and 2 and Clusters 15 and 16, respectively (Fig. 6d and Additional file 4: Table S2). Further analysis of sub-categories suggests that genes involved in transport of peptides and calcium, and genes involved in cell organization, cell division, and cell cycle were rapidly up-regulated or down-regulated in response to BL treatment, respectively (Additional file 4: Table S2; see below).

To identify candidates genes that may be associated with secondary cell wall formation, we detected genes differentially expressed between day 1 (transition into lignin biosynthesis) and day 7 (late phase of lignin biosynthesis) in induced and non-induced suspension cultures. After application of the linear model, 6503 and 8152 genes were identified as differentially expressed according to these criteria (Fig. 6b and Additional file 3: Table S1). Among them, 1273 and 733 genes showed higher or lower expression in induced samples than in non-induced samples during the time course, respectively (Fig. 6b). Functional ontology analysis showed a downregulation of genes involved in cell wall metabolism and an up-regulation of genes involved in the TCA cycle, lipid metabolism, amino acid metabolism, secondary metabolism, and biotic stress response in induced samples on both day 1 and day 7 compared with non-induced samples (Additional file 4: Table S2). Below, we examine specific facets of some of these processes.

### Gene involved in cell wall biogenesis and cell wall modification

#### Expansins

Expansins encode proteins that loosen non-covalent linkages between cellulose microfibrils and are required for cell wall extension [36]. Genes annotated as encoding expansins were differentially expressed in our dataset (Additional file 5: Table S3). For example, genes encoding α-expansin were more highly expressed from day 1 in BL-induced cells, whereas expression of some genes encoding β-expansins decreased and was less than in non-induced samples (Table 1). The differential expression of expansins may reflect their different targets in cell wall modification.

**Table 1:** Expression of switchgrass genes involved in cell wall development

Gene information			Expression information									
Microarray Probe	PvGene	Clade/Gene	Cluster	0 h	Induced				Non-induced		Compare	
					6 h	1 Day	3 Days	7 Days	1 Day	7 Days	1 Day	7 Days
Expansin												
AP13ITG71529_at	Pavir.Aa00711	α-Expansin	4	260	1176	3260	2120	1002	2025	737	*1*	0
KanlowCTG26349_s_at	Pavir.Aa00711	α-Expansin	4	185	572	1927	942	603	1215	397	*1*	0
AP13ITG57935_at	Pavir.Aa00840	β-Expansin	5	3692	3666	134	71	40	16,793	8354	***−*** ***1***	0
AP13CTG27439_s_at	Pavir.Ia02285	β-Expansin	5	2721	5043	2475	440	346	13636	6467	***−*** ***1***	0
CESA												
AP13.12059.m00001_s_at	Pavir.Ca01073	OsCESA1	2	1771	6165	4553	3517	2713	3351	2759	0	0
AP13CTG00607_s_at	Pavir.Ba01088	OsCESA3	4	1160	2574	2285	3019	1894	1391	1362	0	0
AP13CTG25870_s_at	Pavir.J35010	OsCESA8		2702	4804	3832	4513	3535	3877	3669	0	0
KanlowCTG12907_s_at	Pavir.J30974	OsCESA4	16	51	30	98	94	114	58	65	*1*	*1*
KanlowCTG34110_at	Pavir.Ib00804	OsCESA7	0	8	11	9	10	9	9	8	0	0
AP13CTG25049_s_at	Pavir.Bb02205	OsCESA9	0	52	44	56	52	66	56	56	0	0
KanlowCTG15421_s_at	Pavir.Ea00385	OsCESA1	15	1795	1003	1622	2469	1176	1956	1810	0	0
OTHSWCTG06249_s_at		OsCESA5	7	820	904	1810	803	1300	939	1094	*1*	0
AP13CTG06255_s_at	Pavir.J12858	OsCESA2	16	917	549	827	1418	774	941	868	0	0
COBRA												
AP13CTG00322_s_at	Pavir.Ia00526	COB	2	3061	6252	5466	3902	3750	4715	3158	0	0
AP13ITG43381_at	Pavir.Ia00525	COB	5	1771	2628	697	164	697	3370	4215	0	***−*** ***1***
Cellulose-like synthases												
AP13CTG01955_at	Pavir.Ia02025	CSLA	16	204	201	246	269	213	411	224	0	0
AlamoCTG01742_s_at	Pavir.Ib01792	CSLA	12	86	145	279	474	394	207	233	0	*1*
AP13CTG06284RC_s_at	Pavir.Ia00426	CSLC	13	350	151	187	161	144	228	133	0	0
AP13CTG27051_s_at	Pavir.Fa01312	CSLC	5	133	251	39	25	32	147	98	***−*** ***1***	0
KanlowCTG19494_s_at	Pavir.Fb00422	CSLF	5	335	304	327	150	134	154	125	*1*	0
KanlowCTG37527_s_at	Pavir.Ba00688	CSLF	0	164	212	199	147	190	202	174	0	0
Callose synthesis												
KanlowCTG45113_s_at	Pavir.Aa00017	Callose synthase	16	215	100	551	879	379	241	233	*1*	0
AP13ITG74190RC_at	Pavir.Ib02063	Callose-binding	11	64	83	178	116	66	57	48	*1*	0
Cell wall precursor												
KanlowCTG00021_s_at	Pavir.Ia00394	UGD	1	2487	8192	2541	1710	2633	4416	2852	0	0
AP13CTG07489_at	Pavir.Ea03089	UXS	2	416	1017	583	537	605	637	637	0	0
KanlowCTG22514_s_at	Pavir.Ea03089	UXS	0	530	1003	872	820	780	614	813	*1*	0
AP13CTG10419_s_at	Pavir.Gb00500	UXE	1	792	1820	696	695	809	927	934	0	0
AP13CTG28681_s_at	Pavir.Ba03802	UXE	3	186	396	335	329	488	374	300	0	*1*
AP13CTG00023_s_at	Pavir.Gb00638	UXT	4	63	249	409	338	1273	72	156	*1*	*1*
KanlowCTG06720_s_at	Pavir.Ia00627	UXT	2	695	1531	855	1053	1227	588	666	*1*	*1*
AP13CTG08328RC_s_at	Pavir.Ab02692	UER	5	1017	969	566	639	474	2925	1380	***−*** ***1***	0
Arabinoxylan (AX) and glucuronoarabinoxylan (GAX) synthesis (backbone)												
AP13CTG08590_s_at	Pavir.J27018	IRX9	3	280	635	729	481	563	627	441	0	0
AP13ITG73683_s_at	Pavir.Ib01337	IRX9	5	461	385	195	116	124	469	345	***−*** ***1***	***−*** ***1***
AlamoCTG02760_s_at	Pavir.Ea01094	IRX9L	12	136	139	207	370	362	87	174	*1*	*1*
AP13ITG60236_at	Pavir.Ea01094	IRX9L	16	194	153	292	666	548	148	227	*1*	*1*
AP13ITG74502_x_at	Pavir.J37721	IRX14L	1	221	2740	402	234	121	513	208	0	0
KanlowCTG18373_s_at	Pavir.J06597	IRX14L	1	4068	5793	3770	2684	3800	3711	4190	0	0
AP13CTG44601_s_at	Pavir.Ea03872	IRX10	2	388	3191	1018	714	930	609	896	0	0
AP13CTG03198_s_at	Pavir.J03056	IRX10	2	443	3104	851	809	882	1413	1262	***−*** ***1***	***−*** ***1***
Arabinoxylan (AX) and glucuronoarabinoxylan (GAX) synthesis (side chain)												
AP13ITG71461_at	Pavir.Aa01690	GUX	3	74	274	261	122	419	184	226	0	0
KanlowCTG10196_x_at	Pavir.J03459	GUX	1	135	419	170	165	135	353	303	0	0
AP13CTG04376_s_at	Pavir.Aa01745	XAX1	5	146	236	85	73	64	330	217	***−*** ***1***	0
AP13ITG43704_at	Pavir.Ea01415	XAT1	13	136	29	19	15	20	73	53	***−*** ***1***	0
AP13.12340.m00010_s_at	Pavir.Da01068	XAT1	0	179	147	173	190	146	212	227	0	***−*** ***1***
PF02458 (candidates for forming the ester linkages in GAX)												
AP13CTG59034RC_at	Pavir.Gb00939		12	22	34	103	280	171	27	30	0	*1*
AP13ITG73138_at	Pavir.Fa02187		5	190	143	60	27	106	68	135	0	0
KanlowCTG26994RC_at	Pavir.Aa01433		12	73	138	256	626	354	98	105	*1*	*1*
AP13ITG72773_s_at	Pavir.Eb00373		13	1226	269	126	89	117	509	311	***−*** ***1***	0
Pectin biosynthesis												
AP13CTG05511_s_at	Pavir.Ba00640	ARAD1	1	79	95	65	80	75	131	80	***−*** ***1***	0
AP13CTG06439_at	Pavir.Aa00624	GALS1	4	114	422	512	481	490	212	186	*1*	*1*
AP13CTG14035_at	Pavir.J20866	RGXT4	4	129	211	228	254	217	291	219	***−*** ***1***	0
AP13ITG63440_at	Pavir.J14339	RGXT4	4	120	209	247	280	235	304	239	0	0
AP13CTG02428_s_at	Pavir.Ba01601	GAUT1	0	1418	1296	1565	1607	1332	1562	1462	0	0
AP13CTG18174_at	Pavir.Ba00237	GAUT7	2	118	331	247	232	125	447	315	***−*** ***1***	***−*** ***1***
KanlowCTG43804_at	Pavir.Fb01281	GAUT4	16	90	77	138	150	95	206	162	***−*** ***1***	***−*** ***1***
AP13CTG09682_s_at	Pavir.Ab01751	GAUT8	5	609	755	369	449	225	573	371	***−*** ***1***	***−*** ***1***
Pectin esterases												
AP13ITG41170_at	Pavir.Ea01491	PME	5	699	484	73	80	72	104	97	0	0
AP13CTG29622_at	Pavir.Ia02028	PME	13	55	13	7	9	26	12	12	0	0
AP13ITG67053RC_at	Pavir.Eb02306	PAE	5	83	92	27	15	10	602	200	***−*** ***1***	0
AP13CTG10801RC_at	Pavir.Ba00334	PAE	9	838	495	289	407	78	852	353	***−*** ***1***	0
KanlowCTG09579_s_at	Pavir.Eb04062	PAE	1	174	290	170	167	145	280	180	***−*** ***1***	0
Wall structural protein												
AP13CTG23898_at	Pavir.J33924	AGP13	5	923	976	127	126	121	774	399	***−*** ***1***	0
AP13ITG74104_at	Pavir.J33924	AGP13	5	120	158	13	13	16	174	67	***−*** ***1***	***−*** ***1***
AP13ITG53973_s_at	Pavir.Eb02706	FLA	1	315	1924	529	352	111	188	146	*1*	0

Expression values at each data point represent the mean of three biological replicates. Cluster represents the expression groups of filtered genes in induced samples defined by self-organizing map (SOM) method (Fig. 6a). Differential expression genes between induced and non-induced samples on 1 and 7 days were identified by the linear model in LIMMA [84]; − 1 (represented by bold italic), significant lower expression in induced samples than in non-induced samples, 1 (represented by italic), significant higher expression in induced samples than in non-induced samples, 0, no change between induced and non-induced samples

#### Cellulose and COBRA

The cellulose elementary fibril (β-1,4-glucan chain) is synthesized by a complex of cellulose synthase (CESA) proteins [37]. We generated a phylogenetic tree to search for *CESA* genes in switchgrass and analyzed their expression in our dataset (Additional file 1: Figure S5). Expression profiles grouped in Clusters 1 and 2 were observed for the switchgrass genes homologous to rice CESA1, CESA3, and CESA8 involved in primary cell wall biosynthesis. Rice CESA4, CESA7, and CESA 9 are involved in secondary cell wall formation, and gene transcripts homologous to rice CESA4 and CESA9 start to accumulated at 1 day post-treatment with BL, whereas genes homologous to rice CESA7 are slightly induced at 6 h. Considering its higher expression at 1 and 7 days in induced compared to non-induced samples, switchgrass CESA4 may play a major role in cellulose synthesis for the secondary cell wall. qRT-PCR was conducted to further confirm the divergent expression between the CESAs (Additional file 1: Figure S6). Together, our data suggest that primary cell wall cellulose synthesis starts at 6 h and secondary cell wall cellulose synthesis starts at 1 day in induced suspension cultures, with very little secondary cell wall synthesis in non-induced cells.

COB protein encodes a GPI-anchored protein localized in the plasma membrane that facilitates cellulose microfibril organization [38]. One switchgrass *COB* gene ortholog showed a similar expression profile to the *CESA* genes involved in primary cell wall synthesis, whereas another *COB* gene was down-regulated at 1 day post-treatment with BL, but its expression was unchanged in non-induced suspension cultures. We suggest that the protein encoded by the former *COB* gene might guide cellulose deposition in the primary cell wall, whereas that encoded by the latter *COB* gene might somehow inhibit the development of the secondary cell wall.

#### Cellulose synthase-like and callose synthase genes

The CESA super family contains several subfamilies of closely related sequences, termed the cellulose synthase-like (CSL) family [39]. Here we identified 42 switchgrass transcripts homologous to Arabidopsis and rice CSL genes, including *CSLA*, *CSLC*, *CSLD*, *CSLE*, and *CSLF* subfamilies (Additional file 5: Table S3). Two *CSLA* genes, which encode enzymes mediating mannan and glucomannan synthesis [39], were up-regulated, whereas two *CSLCs* involved in xyloglucan biosynthesis [40] were down-regulated in BL-induced cells (Table 1 and Additional file 5: Table S3). The expression of *CSLF*s encoding (1,3;1,4)-β-glucan synthase [40] was unchanged or slightly reduced from 3 days in induced samples. Considering the increased deposition of (1,3;1,4)-β-glucan in induced cells (Fig. 4 and Additional file 1: Figure S2), it is possible that other *CSL* genes are also involved in the synthesis of mixed-linkage glucan in switchgrass cells. For example, *HvCSLH* and the *CSLJ* genes have been identified as being involved in (1,3;1,4)-β-glucan synthesis in wheat [41], but the probes corresponding to their switchgrass homologs were absent from our dataset.

Transcripts encoding a plasmodesmata-localized callose synthase [(1,3)-β-d-glucan synthase] [42] were down-regulated at 6 h and then up-regulated from 1 day, and another gene encoding a plasmodesmata-localized callose-binding protein [43] was up-regulated after 1 day in induced cells. Both genes were more highly expressed at 1 day in induced samples compared with non-induced samples. No consistent pattern of increased callose epitopes was observed in the glycome profiles (Fig. 4 and Additional file 1: Figure S2).

#### Biosynthesis of cell wall precursors

Cell wall polysaccharide precursors are synthesized in the cytoplasm [44, 45], and in the Golgi [46]. Many genes involved in the synthesis of cell wall precursors were identified in our dataset (Table 1 and Additional file 5: Table S3). Differential expression patterns were observed for genes encoding enzymes involved in the formation of eleven nucleoside diphosphate (NDP)-sugars (UDP-Gal, UDP-Glc, UDP-Rha, UDP-GlcA, UDP-GalA, UDP-Xyl, UDP-apiose, UDP-Ara, GDP-Man, GDP-Glc, and GDP-Fuc) (Additional file 1: Figure S7). Generally speaking, transcripts encoding enzymes involved in the last steps of UDP-Rha, UDP-Gal, and UDP-GalA synthesis decreased, whereas transcripts encoding enzymes involved in the synthesis of UDP-Xyl [47] and UDP-Ara [44] increased after BL treatment (Additional file 1: Figure S7 and Additional file 5: Table S3). Consistently, genes that encode transporters of cytosolic UDP-xylose into the Golgi (UXT, UDP-xylose transporter) [48] were up-regulated in induced cells. The elevated expression of UXS (UDP-xylose synthase), UXE (UDP-xylose epimerase), and UXT genes and decreased expression of the UER (nucleotide-rhamnose synthase/epimerase-reductase) gene that encodes an enzyme for the final step in the biosynthesis of UDP-Rha [49] at 1 and 7 days (Table 1 and Additional file 1: Figure S7) may underlie the BL-induced decreases in rhamnose and arabinose/xylose ratio of the cell wall residues (Fig. 2).

#### Arabinoxylan (AX) and glucuronoarabinoxylan (GAX) synthesis

A large number of arabinosyl (Ara) and/or glucuronosyl (GlcA) side chains are attached to the xylan backbone to form arabinoxylan and glucuronoarabinoxylan (GAX) in grass cell walls [50]. Several members of the GT43 and GT47 families, such as IRX9, IRX10, and IRX14, have been shown to be involved in xylan backbone extension in Arabidopsis [50–52]. Genes homologous to IRX10 and IRX14 were co-induced in switchgrass cells in response to BL, but genes homologous to IRX9/IRX9L were differentially expressed. Considering that IRX10L and IRX14, rather than IRX9, are required for xylan backbone synthesis in primary cell walls of Arabidopsis [53], the differential expression of these GT43 and GT47 genes in switchgrass may suggest differential functions in xylan extension in the primary and/or secondary cell wall.

A few genes in the GT8 family (GUX1 and GUX2 in Arabidopsis) [54] and GT61 family (XAT1 in wheat and XAX1 in rice) [55] have been identified to decorate the xylan backbone with glucuronosyl and arabinosyl substitution, respectively. An increased expression of switchgrass homologs of GUX1 was observed in BL-induced cells. However, the expression of switchgrass homologs of XAT1 and XAX1 was decreased in induced cells, with a lower value on day 1 and/or day 7 compared with non-induced cells.

Arabinoxylan and glucuronoarabinoxylan may be ester-linked to ferulic and *p*-coumaric acids in grass cell walls, providing a barrier to cell wall digestibility [56]. Enzymes in the Pfam family PF02458 are considered as candidates for forming the ester linkages in GAX [56, 57]. We examined the expression profiles of genes encoding enzymes belonging to the PF02458 family in our dataset (Additional file 5: Table S3), and further selected candidate genes according to the differential accumulation of wall-esterified ferulic acid (decreased) and *p*-coumaric acid (increased) during the time course in induced samples (Table 1). Expression of gene AP13ISTG72773 was down-regulated in induced cells (Additional file 5: Table S3), whereas P13CTG59034-RC and KanlCTG26994-RC were up-regulated. These two genes could be candidate targets for reducing the degree of ester linkages in the GAX component of grass cell walls for increasing the efficiency of conversion of biomass to sugars.

#### Pectin and cell wall structural proteins

A limited number of enzymes have been unambiguously identified to be specifically required for synthesis of pectic polysaccharides [58, 59]. The switchgrass homolog of RGXT that is responsible for the xylosyl-linkage in RG-II [60] was weakly induced in the cell cultures in response to BL. Arabidopsis ARAD1 [61] and GALS1 [62] have been shown to encode enzymes involved in attaching α-1,5-arabinans and β-1,4-galactans onto the RG-I backbone, respectively. The switchgrass homologs of ARAD1 show overall a slight up-regulation in response to BL, but are expressed at lower levels than in non-induced samples on day 1 (Table 1). Interestingly, one homolog of GALS1 showed elevated expression throughout the time course in induced suspension cultures, with no change in expression on day 1 and day 7 in non-induced cultures. Considering the fact that β-1,4-galactans are more abundant in secondary than in primary cell walls [62], we suggest that this gene is a candidate for involvement in the synthesis of the galactan side chain of RG-I.

Several members of the galacturonosyltransferase (GAUT) family are responsible for constructing the HG chain [63]. Switchgrass genes belonging to the GAUT family were expressed with varying patterns in induced suspension cells, but were expressed lower at day 1 and day 7 than in non-induced cells. GAUT1 expression was relatively unaffected by BL addition, whereas the expression of GAUT7 peaked at 6 h post BL addition. Considering that Arabidopsis GAUT1 and GAUT7 form a core complex for HG synthesis [63], we suggest that a peak of HG synthesis might occur at 6 h and then decrease in induced samples to an overall lower level than in non-induced samples.

The largely unbranched HG polymer may be modified by methylesterification at C-6, and acetylation at O-2 and O-3, of GalA residues [64]. Most pectin methylesterase (PME) genes were down-regulated in both induced and non-induced cell cultures, consistent with the low degree of pectin methylation detected by glycome profiling (Fig. 4). Pectin acetylesterase (PAE) lowers the acetylation status of pectic polymers [65]. We have listed several PAE candidates which are down-regulated in BL-induced cell but up-regulated or unaffected on days 1 and 7 in non-induced cultures (Table 1).

We also observed differential expression of genes encoding cell wall structural proteins (Additional file 5: Table S3). For example, a rapid induction by 6 h and a higher expression on day 1 was found for genes encoding fasciclin-like arabinogalactan proteins (FLA) in induced compared with non-induced cells (Table 1 and Additional file 5: Table S3). FLAs are plasma membrane-bound proteins with signal peptides that are involved in secondary cell wall development in Arabidopsis and rice [66]. We suggest that these FLAs may have a similar function in cell membrane signaling in secondary cell wall formation in switchgrass suspension cells. Interestingly, expression of two genes encoding arabinogalactan proteins (AGPs) involved in cell–cell signaling [67] was significantly down-regulated by day 1 in induced cells, and these genes were expressed at lower levels at day 1 and/or day 7 than in non-induced cells (Table 1). We suggest that these *AGP* genes might be involved in negative regulation of secondary cell wall formation in switchgrass suspension cultures.

#### Transcriptional control of S lignin biosynthesis

Twelve enzymes are well known to be involved in plant monolignol biosynthesis, and we have previously identified a full suite of candidate switchgrass genes potentially involved in this pathway [11]. Here, we have confirmed the abundance of S lignin in BL-induced suspension cells. Genes encoding phenylalanine ammonia-lyase (PAL), cinnamate 4-hydroxylase (C4H), 4-coumarate:CoA ligase (4CL), and caffeoyl CoA 3-*O*-methyltransferase (CCoAoMT) were strongly up-regulated in BL-induced cells. A coordinated accumulation of transcripts encoding cinnamoyl CoA reductase (CCR), caffeic acid 3-*O*-methyltransferase (COMT), and p-coumaroyl-CoA:monolignol transferase (PMT) was observed in induced cells after day 1. Cinnamyl alcohol dehydrogenase (CAD) and ferulate/coniferaldehyde 5-hydroxylase (F5H) were the most highly expressed lignin synthesis genes during the time course in induced samples. Based on these results, a preferred route for S unit biosynthesis in induced cells is suggested in Additional file 1: Figure S8.

**Fig. 7:**
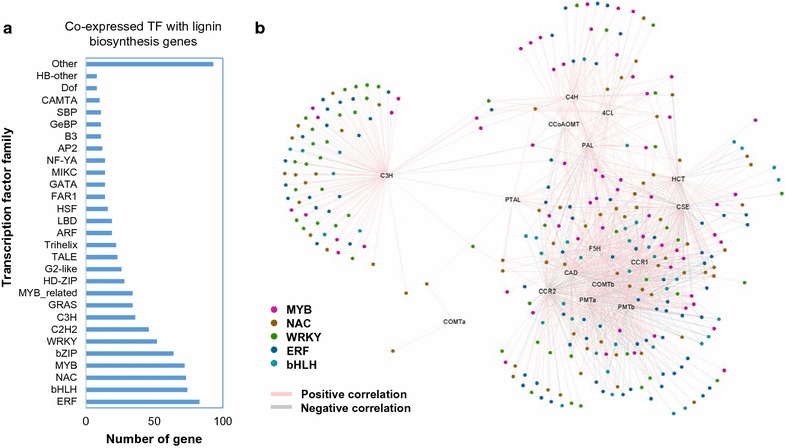
Co-expression analysis of transcription factors with lignin biosynthesis genes. **a** Numbers of TFs of different families that are co-expressed with lignin biosynthesis genes. **b** Co-expression network of TFs and lignin biosynthesis genes. Pink and gray lines indicate positive and negative correlations, respectively

The differential expression of lignin biosynthesis genes suggests control by multiple transcription factors (TFs). To identify candidate TFs involved in S lignin accumulation, co-expression analysis was applied to our dataset using Pearson’s correlation method and lignin biosynthesis genes as bait genes (Additional file 6: Table S4). Most TF genes that were co-expressed with lignin biosynthesis genes belonged to the ERF, bHLH, NAC, MYB, bZIP, and WRKY families (Fig. 7). Several members of the ERF, NAC, MYB, and WRKY families have already been characterized for regulation of lignin genes [68–71], and are present in our candidate TF list. We found a group of TFs that were co-expressed with CCR, COMT, ferulate/coniferaldehyde 5-hydroxylase (F5H), and CAD (all potentially specific for S lignin biosynthesis), whereas a separate group of TFs showed co-expression with PAL, C4H, 4CL, and CCoAoMT (Fig. 7). Interestingly, caffeoyl shikimate 3′-hydroxylase (C3ˊH) was an outlier, suggesting independent regulation by additional TFs (Fig. 7).

#### Genes associated with stress and signaling

In addition, we identified candidate genes involved in hormone signaling and metabolism, and biotic stress. Details are provided and discussed in Additional file 7: Results and Discussion and Additional file 8: Table S7. In our BL-induced suspension cultures, we detected increased levels of lignin without tracheary element formation, and the elevated expression of many genes involved in sensing and resistance to pathogen attack (Additional file 5: Table S3 and Additional file 9: Table S5) which did not occur in non-induced cells. This suggests a role for BR in the activation of lignin biosynthesis as a defense response, rather than as part of a developmentally regulated program of cellular differentiation. The response of the switchgrass cells to BR appears similar to the response of Arabidopsis cell cultures to methyl JA, which includes increased production of monolignols and up-regulation of defense genes [72]. We suggest that the triple effects of BR signaling on immune responses and phenylpropanoid metabolism might not be independent events.

## Conclusions

Improving the conversion efficiency of lignocellulosic biomass to fuels requires a better understanding of cell wall properties. However, until now, a relatively limited number of cell wall-related genes have been manipulated in switchgrass; these include UAM, GAUT, COMT, CCR, hydroxycinnamoyl CoA shikimate:quinate hydroxycinnamoyl transferase (HCT), and the transcription factors MYB4 and MYB46 [11, 68, 69, 73–78]. We have established a switchgrass suspension cell system in which lignification can be robustly activated by exogenously supplied brassinolide. Biochemical analysis of cell wall composition and associated transcript profiling has provided a resource to identify genes related to both primary and secondary cell wall processes.

**Fig. 8:**
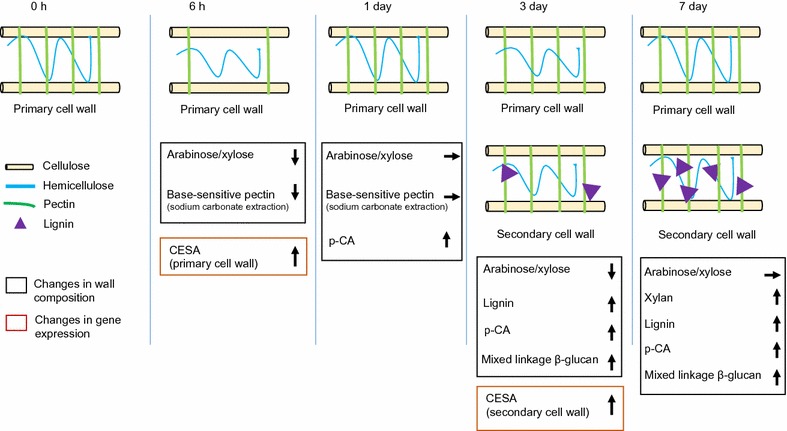
Model for BL-mediated cell wall development in switchgrass suspension cultures. The cell wall components cellulose, hemicellulose, pectin, and lignin are represented as yellow, blue, and green lines, and triangles symbols, respectively. Dynamic changes in cell wall composition and gene expression are represented in black and red squares, respectively. The direction of arrows shows increase (↑), restoration (→), or decrease (↓) in content of the corresponding components. Significant changes in cell wall composition and gene expression at each time point are as follows: at 6 h, a decrease in arabinose/xylose ratio, contents of pectin released by sodium carbonate, and an up-regulation of CESAs involved in primary cell wall synthesis; at 1 day, restored content of arabinose, xylose, and pectin; at 3 days, an increased content of lignin, p-CA and mixed-linkage β-glucan, a reduced ratio of arabinose/xylose, and an up-regulation of CESAs associated with secondary cell wall synthesis; at 7 days, an increased content of xylan, lignin, p-CA and mixed-linkage β-glucan, and restored ratio of arabinose/xylose

Based on our data, we present a model for key biochemical and transcriptional events associated with BL-induced reprogramming of cell wall development in switchgrass suspension cultures (Fig. 8). As early as 6 h after BL treatment, there is a reduced ratio of arabinose to xylose and a reduced content of base-sensitive pectic material released by sodium carbonate. At the same time, genes encoding CESAs associated with the primary cell wall are up-regulated. The low ratio of arabinose to xylose reflects a high degree of linear xylans, which have reduced crosslinking with cellulose and other components of the wall [54, 79]. These changes suggest an early stimulation of primary cell wall synthesis.

By 1 day after BL treatment, the content of arabinose, xylose, and pectin increases, as does the ratio of wall-bound phenolic acids (p-CA/FA), suggesting initiation of secondary wall synthesis. After 3 days of BL treatment, there is an accumulation of xylans, lignin, p-CA and mixed-linkage β-glucans, as well as a reduction in the arabinose/xylose ratio. The expression of CESA forms associated with secondary cell wall increases. We suggest that secondary cell wall development is occurring in the cells within 3 days of BL addition.

Seven days after BL treatment, the initial ratio of arabinose to xylose is restored, and elevated levels of xylans, mixed-linkage glucan, and lignin are deposited in the cell walls. These changes correlate with reduced expression of genes functioning in glycolysis, fermentation, and cell wall biosynthesis and increased expression of genes functioning in amino acid, nucleotide, and secondary metabolism. At later times of cell wall lignification, carbon flow might be shifted from formation of structural polymers to formation of storage polymers and secondary metabolites in suspension cells.

The identification of genes associated with the above program of cell wall biogenesis provides a basis for application for high throughput genetic engineering of biofuel crops to reduce biomass recalcitrance and enhance bioenergy production.

## Methods

### Determination of polysaccharide composition

Cell wall extraction and polysaccharide estimation were as described previously [7, 26]. Briefly, the alcohol-insoluble residue (AIR) of cell walls from suspension cells was extracted with water and then alcohol. After hydrolysis with 2 M trifluoroacetic acid (TFA), the concentrations of cell wall matrix polysaccharide components (arabinose, xylose, mannose, galactose, rhamnose, fucose, and glucose) were determined by gas chromatography–mass spectrometry (GC–MS) as the corresponding alditol acetate derivatives in comparison to authentic standards.

### Cell wall fractionalization and glycome profiling

Sequential cell wall extraction and subsequent glycome profiling were performed as described previously [7, 26]. Samples were sequentially extracted with 50 mM ammonium oxalate (pH ~ 5.0), 50 mM sodium carbonate with 0.5% (w/v) sodium borohydride (pH ~ 10), 1 M KOH with 1% (w/v) sodium borohydride, 4 M KOH with 1% (w/v) sodium borohydride, chlorite, and 4 M KOH with 1% (w/v) sodium borohydride post-chlorite. The supernatants were neutralized, dialyzed, lyophilized, and saved after each fractionation step [26]. The information for each plant glycan-directed antibody used in subsequent ELISA assays is available in previous publications [26, 80] and in a web database (http://www.wallmabdb.net).

### Determination of lignin content and composition

Switchgrass stems (at the E4I2 and E4I4 stages) and suspension cells were collected and prepared as described previously [11, 81]. Lignin content and composition were determined by thioacidolysis followed by gas chromatography/mass spectrometry (GC/MS) of trimethylsilyl derivatives [11]. The extraction of soluble phenolics using 50% (v/v) methanol and 1.5% (v/v) acetic acid was as previously described [68]. Esterified cell wall-bound phenolics were subjected to low-temperature alkaline hydrolysis and determined by high-performance liquid chromatography (HPLC) [81].

### Nuclear magnetic resonance (NMR) analysis

Lignin samples from switchgrass stems at the E4I4 stage and induced suspension cells at day 7 were isolated and 2D HSQC NMR spectral analysis was carried out as described previously [74].

### Gene expression profiling, clustering, and annotation

Microarray analysis was conducted on induced samples at 0, 6 h, 1, 3, and 7 days and on non-induced samples at 1 and 7 days with replicates as previously described [11]. The raw and normalized microarray data for the present suspension cell samples can be downloaded at the Switchgrass Functional Genomics Server (http://switchgrassgenomics.noble.org) [82]. The microarray probes were mapped to *Panicum virgatum* v1.1 reference genome (http://phytozome.jgi.doe.gov/) using BLAST [83]. Gene expression patterns were clustered by principal component analysis (PCA), self-organizing map (SOM) (https://cran.r-project.org/web/packages/som/index.html), and the linear model (Limma) [84] using R 3.0.1. Gene classification and transcription factor (TF) identification in switchgrass was based on the Arabidopsis and rice homologs as described previously [85]. Functional classification of differentially expressed genes was performed as described in Additional file 2: Materials and Methods.

### RNA isolation and real-time PCR

The methods for RNA isolation, cDNA synthesis, primer design, and qRT-PCR analysis were performed as previously described [11]. Sequences of primers are provided in Additional file 10: Table S6. Transcript levels were determined by relative quantification using the ubiquitin gene as reference.

## Additional files

**Additional file 1: Figure S1.** Comparison of the monosaccharide composition of switchgrass suspension cell walls with those of other plant cell suspensions. **Figure S2.** Glycome profiling heat map showing the relative abundance of cell wall glycan epitopes released sequentially from the AIR fractions of non-induced and BL-induced suspension cells. **Figure S3**. Measurement of wall-bound phenolic acids in non-induced and BL-induced switchgrass suspension culture cells. **Figure S4.** Principal component analysis (PCA) of genes expressed in BL-induced cultures. **Figure S5.** The expression of CESA genes in non-induced and BL-induced cultures. **Figure S6**. Validation of cell wall-related gene expression in switchgrass suspension cultures by qRT-PCR. **Figure S7.** Overview of changes in transcript levels for genes involved in cell wall precursor synthesis in BL-induced cultures. **Figure S8**. Proposed route for S lignin biosynthesis in BL-induced suspension cells.

**Additional file 2: Materials and Methods.** Supplementary information on pre-processing of the microarray dataset.

**Additional file 3: Table S1.** List of differentially expressed genes in switchgrass suspension cells using SOM and the linear model.

**Additional file 4: Table S2.** Functional distribution of differentially expressed genes in switchgrass suspension cells.

**Additional file 5: Table S3.** Cell wall-related functional groups in switchgrass suspension cells.

**Additional file 6: Table S4.** List of TFs co-expressed with lignin biosynthesis genes in switchgrass suspension cells.

**Additional file 7: Results and Discussion.** Supplementary information on genes involved in hormone signaling and metabolism, and biotic stress.

**Additional file 8: Table S7.** Expression of switchgrass genes involved in hormone signaling.

**Additional file 9: Table S5.** Expression of switchgrass genes involved in biotic stress responses.

**Additional file 10: Table S6.** Sequences of qRT-PCR primers.

**Additional file 11: Table S8.** The Student’s *t*-tests on the monosaccharide composition and sequential extraction between pairwise samples.

## References

[1] Vogel J (2008). Unique aspects of the grass cell wall. Curr Opin Plant Biol.

[2] Loque D, Scheller HV, Pauly M (2015). Engineering of plant cell walls for enhanced biofuel production. Curr Opin Plant Biol.

[3] Pauly M, Keegstra K (2010). Plant cell wall polymers as precursors for biofuels. Curr Opin Plant Biol.

[4] Himmel ME, Ding S-Y, Johnson DK, Adney WS, Nimlos MR, Brady JW (2007). Biomass recalcitrance: engineering plants and enzymes for biofuels production. Science.

[5] Bouton JH (2007). Molecular breeding of switchgrass for use as a biofuel crop. Curr Opin Genet Dev.

[6] Cosgrove DJ, Jarvis MC (2012). Comparative structure and biomechanics of plant primary and secondary cell walls. Front Plant Sci.

[7] Pattathil S, Hahn MG, Dale BE, Chundawat SP (2015). Insights into plant cell wall structure, architecture, and integrity using glycome profiling of native and AFEXTM-pre-treated biomass. J Exp Bot.

[8] Handakumbura PP, Hazen SP (2012). Transcriptional regulation of grass secondary cell wall biosynthesis: playing catch-up with *Arabidopsis thaliana*. Front Plant Sci..

[9] Grabber JH, Ralph J, Lapierre C, Barrière Y (2004). Genetic and molecular basis of grass cell-wall degradability. I. Lignin–cell wall matrix interactions. CR Biol.

[10] Barros J, Serrani-Yarce JC, Chen F, Baxter D, Venables BJ, Dixon RA (2016). Role of bifunctional ammonia-lyase in grass cell wall biosynthesis. Nat Plants..

[11] Shen H, Mazarei M, Hisano H, Escamilla-Trevino L, Fu CX, Pu YQ (2013). A genomics approach to deciphering lignin biosynthesis in switchgrass. Plant Cell..

[12] Kulkarni AR, Pattathil S, Hahn MG, York WS, O’Neill MA (2012). Comparison of arabinoxylan structure in bioenergy and model grasses. Ind Biotechnol.

[13] Sarath G, Baird LM, Vogel KP, Mitchell RB (2007). Internode structure and cell wall composition in maturing tillers of switchgrass (*Panicum virgatum* L.). Biores Technol.

[14] Mustafa NR, de Winter W, van Iren F, Verpoorte R (2011). Initiation, growth and cryopreservation of plant cell suspension cultures. Nat Protocols.

[15] Burke D, Kaufman P, McNeil M, Albersheim P (1974). The structure of plant cell walls: VI. A survey of the walls of suspension-cultured monocots. Plant Physiol.

[16] Karkonen A, Koutaniemi S (2010). Lignin biosynthesis studies in plant tissue cultures. J Integr Plant Biol.

[17] Creelman RA, Mullet JE (1997). Oligosaccharins, brassinolides, and jasmonates: nontraditional regulators of plant growth, development, and gene expression. Plant Cell..

[18] Yamamoto R, Fujioka S, Demura T, Takatsuto S, Yoshida S, Fukuda H (2001). Brassinosteroid levels increase drastically prior to morphogenesis of tracheary elements. Plant Physiol.

[19] Negi S, Tak H, Ganapathi TR (2015). In vitro xylem vessel elements formation from banana embryogenic cells and expression analysis of vessel development-related genes. Plant Biotechnol Rep..

[20] Oda Y, Mimura T, Hasezawa S (2005). Regulation of secondary cell wall development by cortical microtubules during tracheary element differentiation in Arabidopsis cell suspensions. Plant Physiol.

[21] Kubo M, Udagawa M, Nishikubo N, Horiguchi G, Yamaguchi M, Ito J (2005). Transcription switches for protoxylem and metaxylem vessel formation. Genes Dev.

[22] Mazarei M, Al-Ahmad H, Rudis MR, Joyce BL, Stewart CN (2011). Switchgrass (*Panicum virgatum* L.) cell suspension cultures: establishment, characterization, and application. Plant Sci.

[23] Eberhardt TL, Bernards MA, He L, Davin LB, Wooten JB, Lewis NG (1993). Lignification in cell suspension cultures of *Pinus taeda*. In situ characterization of a gymnosperm lignin. J Biol Chem.

[24] Labourel A, Crouch LI, Brás JLA, Jackson A, Rogowski A, Gray J (2016). The mechanism by which arabinoxylanases can recognize highly decorated xylans. J Biol Chem.

[25] Bar-Peled M, Urbanowicz BR, O’Neill MA (2012). The synthesis and origin of the pectic polysaccharide rhamnogalacturonan II—insights from nucleotide sugar formation and diversity. Front Plant Sci..

[26] Pattathil S, Avci U, Miller JS, Hahn MG, Himmel ME (2012). Immunological approaches to plant cell wall and biomass characterization: glycome profiling. Biomass conversion: methods and protocols.

[27] Pilnik W, Rombouts FM (1985). Polysaccharides and food processing. Carbohydr Res.

[28] Brummell DA (2006). Cell wall disassembly in ripening fruit. Funct Plant Biol.

[29] Merali Z, Collins SRA, Elliston A, Wilson DR, Käsper A, Waldron KW (2015). Characterization of cell wall components of wheat bran following hydrothermal pretreatment and fractionation. Biotechnol Biofuels.

[30] Hedley CL (2000). Carbohydrates in grain legume seeds: improving nutritional quality and agronomic characteristics.

[31] Schmidt D, Schuhmacher F, Geissner A, Seeberger PH, Pfrengle F (2015). Automated synthesis of arabinoxylan-oligosaccharides enables characterization of antibodies that recognize plant cell wall glycans. Chemistry.

[32] Ralph J, Grabber JH, Hatfield RD (1995). Lignin-ferulate cross-links in grasses: active incorporation of ferulate polysaccharide esters into ryegrass lignins. Carbohydr Res.

[33] Grabber JH, Ralph J, Hatfield RD (2000). Cross-linking of maize walls by ferulate dimerization and incorporation into lignin. J Agric Food Chem.

[34] Chen F, Duran AL, Blount JW, Sumner LW, Dixon RA (2003). Profiling phenolic metabolites in transgenic alfalfa modified in lignin biosynthesis. Phytochemistry.

[35] Thimm O, Blasing O, Gibon Y, Nagel A, Meyer S, Kruger P (2004). MAPMAN: a user-driven tool to display genomics data sets onto diagrams of metabolic pathways and other biological processes. Plant J..

[36] Yennawar NH, Li LC, Dudzinski DM, Tabuchi A, Cosgrove DJ (2006). Crystal structure and activities of EXPB1 (Zea m 1), alpha, beta-expansin and group-1 pollen allergen from maize. Proc Natl Acad Sci USA.

[37] Kumar M, Turner S (2015). Plant cellulose synthesis: CESA proteins crossing kingdoms. Phytochemistry.

[38] Sorek N, Sorek H, Kijac A, Szemenyei HJ, Bauer S, Hématy K (2014). The Arabidopsis COBRA protein facilitates cellulose crystallization at the plasma membrane. J Biol Chem.

[39] Liepman AH, Cavalier DM (2012). The cellulose synthase-like A and cellulose synthase-like C families: recent advances and future perspectives. Front Plant Sci..

[40] Schwerdt JG, MacKenzie K, Wright F, Oehme D, Wagner JM, Harvey AJ (2015). Evolutionary dynamics of the cellulose synthase gene superfamily in grasses. Plant Physiol.

[41] Fincher GB (2009). Revolutionary times in our understanding of cell wall biosynthesis and remodeling in the grasses. Plant Physiol.

[42] Yadav SR, Yan D, Sevilem I, Helariutta Y (2014). Plasmodesmata-mediated intercellular signaling during plant growth and development. Specialised membrane domains of plasmodesmata, plant intercellular nanopores. Front Plant Sci.

[43] Simpson C, Thomas C, Findlay K, Bayer E, Maule AJ (2009). An Arabidopsis GPI-anchor plasmodesmal neck protein with callose binding activity and potential to regulate cell-to-cell trafficking. Plant Cell..

[44] Alonso AP, Piasecki RJ, Wang Y, LaClair RW, Shachar-Hill Y (2010). Quantifying the labeling and the levels of plant cell wall precursors using ion chromatography tandem mass spectrometry. Plant Physiol.

[45] Fry SC (2001). Plant cell wall biosynthesis, in eLS.

[46] Bar-Peled M, O’Neill MA (2011). Plant nucleotide sugar formation, interconversion, and salvage by sugar recycling. Annu Rev Plant Biol.

[47] Harper AD, Bar-Peled M (2002). Biosynthesis of UDP-xylose. Cloning and characterization of a novel Arabidopsis gene family, UXS, encoding soluble and putative membrane-bound UDP-glucuronic acid decarboxylase isoforms. Plant Physiol.

[48] Ebert B, Rautengarten C, Guo X, Xiong G, Stonebloom S, Smith-Moritz AM (2015). Identification and characterization of a golgi-localized UDP-xylose transporter family from Arabidopsis. Plant Cell..

[49] Han X, Qian L, Zhang L, Liu X (2015). Structural and biochemical insights into nucleotide–rhamnose synthase/epimerase–reductase from *Arabidopsis thaliana*. Biochim Biophys Acta Proteins Proteom..

[50] Scheller HV, Ulvskov P (2010). Hemicelluloses. Annu Rev Plant Biol.

[51] Urbanowicz BR, Peña MJ, Moniz HA, Moremen KW, York WS (2014). Two Arabidopsis proteins synthesize acetylated xylan in vitro. Plant J..

[52] Jensen JK, Johnson NR, Wilkerson CG (2014). *Arabidopsis thaliana* IRX10 and two related proteins from *Psyllium* and *Physcomitrella patens* are xylan xylosyltransferases. Plant J..

[53] Mortimer JC, Faria-Blanc N, Yu X, Tryfona T, Sorieul M, Ng YZ (2015). An unusual xylan in Arabidopsis primary cell walls is synthesised by GUX3, IRX9L, IRX10L and IRX14. Plant J..

[54] Mortimer JC, Miles GP, Brown DM, Zhang ZN, Segura MP, Weimar T (2010). Absence of branches from xylan in Arabidopsis gux mutants reveals potential for simplification of lignocellulosic biomass. Proc Natl Acad Sci USA.

[55] Anders N, Wilkinson MD, Lovegrove A, Freeman J, Tryfona T, Pellny TK (2012). Glycosyl transferases in family 61 mediate arabinofuranosyl transfer onto xylan in grasses. Proc Natl Acad Sci USA.

[56] Mitchell RAC, Dupree P, Shewry PR (2007). A novel bioinformatics approach identifies candidate genes for the synthesis and feruloylation of arabinoxylan. Plant Physiol.

[57] Bartley LE, Peck ML, Kim S-R, Ebert B, Manisseri C, Chiniquy DM (2013). Overexpression of a BAHD acyltransferase, OsAt10, alters rice cell wall hydroxycinnamic acid content and saccharification. Plant Physiol.

[58] Harholt J, Suttangkakul A, Scheller HV (2010). Biosynthesis of pectin. Plant Physiol.

[59] Atmodjo MA, Hao Z, Mohnen D (2013). Evolving views of pectin biosynthesis. Annu Rev Plant Biol.

[60] Liu X-L, Liu L, Niu Q-K, Xia C, Yang K-Z, Li R (2011). MALE GAMETOPHYTE DEFECTIVE 4 encodes a rhamnogalacturonan II xylosyltransferase and is important for growth of pollen tubes and roots in Arabidopsis. Plant J..

[61] Harholt J, Jensen JK, Sørensen SO, Orfila C, Pauly M, Scheller HV (2006). ARABINAN DEFICIENT 1 Is a putative arabinosyltransferase involved in biosynthesis of pectic arabinan in Arabidopsis. Plant Physiol.

[62] Liwanag AJM, Ebert B, Verhertbruggen Y, Rennie EA, Rautengarten C, Oikawa A (2012). Pectin biosynthesis: GALS1 in *Arabidopsis thaliana* is a β-1, 4-galactan β-1, 4-galactosyltransferase. Plant Cell..

[63] Atmodjo MA, Sakuragi Y, Zhu X, Burrell AJ, Mohanty SS, Atwood JA (2011). Galacturonosyltransferase (GAUT)1 and GAUT7 are the core of a plant cell wall pectin biosynthetic homogalacturonan:galacturonosyltransferase complex. Proc Natl Acad Sci USA.

[64] Caffall KH, Pattathil S, Phillips SE, Hahn MG, Mohnen D (2009). *Arabidopsis thaliana* T-DNA mutants implicate GAUT genes in the biosynthesis of pectin and xylan in cell walls and seed testa. Mol Plant..

[65] de Souza A, Hull PA, Gille S, Pauly M (2014). Identification and functional characterization of the distinct plant pectin esterases PAE8 and PAE9 and their deletion mutants. Planta.

[66] Oikawa A, Joshi HJ, Rennie EA, Ebert B, Manisseri C, Heazlewood JL (2010). An Integrative approach to the identification of Arabidopsis and Rrce genes involved in xylan and secondary wall development. PLoS ONE.

[67] Guan Y, Nothnagel EA (2004). Binding of arabinogalactan proteins by Yariv phenylglycoside triggers wound-like responses in Arabidopsis cell cultures. Plant Physiol.

[68] Shen H, He X, Poovaiah CR, Wuddineh WA, Ma J, Mann DGJ (2012). Functional characterization of the switchgrass (*Panicum virgatum*) R2R3-MYB transcription factor PvMYB4 for improvement of lignocellulosic feedstocks. New Phytol.

[69] Zhong R, Yuan Y, Spiekerman JJ, Guley JT, Egbosiuba JC, Ye Z-H (2015). Functional characterization of NAC and MYB transcription factors Involved in regulation of biomass production in switchgrass (*Panicum virgatum*). PLoS ONE.

[70] Zhao Q, Dixon RA (2011). Transcriptional networks for lignin biosynthesis: more complex than we thought?. Trends Plant Sci.

[71] Hussey SG, Mizrachi E, Creux NM, Myburg AA (2013). Navigating the transcriptional roadmap regulating plant secondary cell wall deposition. Front Plant Sci..

[72] Pauwels L, Morreel K, De Witte E, Lammertyn F, Van Montagu M, Boerjan W (2008). Mapping methyl jasmonate-mediated transcriptional reprogramming of metabolism and cell cycle progression in cultured Arabidopsis cells. Proc Natl Acad Sci USA.

[73] Escamilla-Treviño LL, Shen H, Hernandez T, Yin Y, Xu Y, Dixon RA (2014). Early lignin pathway enzymes and routes to chlorogenic acid in switchgrass (*Panicum virgatum* L.). Plant Mol Biol.

[74] Shen H, Poovaiah CR, Ziebell A, Tschaplinski TJ, Pattathil S, Gjersing E (2013). Enhanced characteristics of genetically modified switchgrass (*Panicum virgatum* L.) for high biofuel production. Biotechnol for Biofuels..

[75] Fu C, Mielenz JR, Xiao X, Ge Y, Hamilton CY, Rodriguez M (2011). Genetic manipulation of lignin reduces recalcitrance and improves ethanol production from switchgrass. Proc Natl Acad Sci USA.

[76] Dumitrache A, Natzke J, Rodriguez M, Yee KL, Thompson OA, Poovaiah CR (2017). Transgenic switchgrass (Panicum virgatum L.) targeted for reduced recalcitrance to bioconversion: a two-year comparative analysis of field-grown lines modified for target gene or genetic element expression. Plant Biotechnol J..

[77] Willis J, Smith J, Mazarei M, Zhang J, Turner G, Decker S (2016). Downregulation of the UDP-arabinomutase gene in switchgrass *(Panicum virgatum* L.) results in increased cell wall lignin while reducing arabinose-glycans. Front Plant Sci..

[78] Escamilla-Treviño LL, Shen H, Uppalapati SR, Ray T, Tang Y, Hernandez T (2010). Switchgrass (*Panicum virgatum*) possesses a divergent family of cinnamoyl CoA reductases with distinct biochemical properties. New Phytol.

[79] Brice RE, Morrison IM (1982). The degradation of isolated hemicelluloses and lignin-hemicellulose complexes by cell-free, rumen hemicellulases. Carbohydr Res.

[80] Pattathil S, Avci U, Baldwin D, Swennes AG, McGill JA, Popper Z (2010). A comprehensive toolkit of plant cell wall glycan-directed monoclonal antibodies. Plant Physiol.

[81] Shen H, Fu C, Xiao X, Ray T, Tang Y, Wang Z (2009). Developmental control of lignification in stems of lowland switchgrass variety Alamo and the effects on saccharification efficiency. BioEnergy Res..

[82] Zhang JY, Lee YC, Torres-Jerez I, Wang M, Yin Y, Chou WC (2013). Development of an integrated transcript sequence database and a gene expression atlas for gene discovery and analysis in switchgrass (*Panicum virgatum* L.). Plant J..

[83] Chen X, Ma Q, Rao X, Tang Y, Wang Y, Li G (2015). Genome-scale identification of cell-wall-related genes in switchgrass through comparative genomics and computational analyses of transcriptomic data. BioEnergy Res..

[84] Ritchie ME, Phipson B, Wu D, Hu Y, Law CW, Shi W (2015). limma powers differential expression analyses for RNA-sequencing and microarray studies. Nucleic Acids Res.

[85] Rao X, Lu N, Li G, Nakashima J, Tang Y, Dixon RA (2016). Comparative cell-specific transcriptomics reveals differentiation of C4 photosynthesis pathways in switchgrass and other C4 lineages. J Exp Bot.

