# Supplementary material for: Dynamic changes in transcriptome and cell wall composition underlying brassinosteroid-mediated lignification of switchgrass suspension cells
Source: Biotechnol Biofuels. 2017 Nov 30;10:266. doi: 10.1186/s13068-017-0954-2 (PMC5707915; doi:10.1186/s13068-017-0954-2)
Supplement: Supplementary file 1 — Additional file 1: Figure S1. Comparison of the monosaccharide composition of switchgrass suspension cell walls with those of other plant cell suspensions. Figure S2. Glycome profiling heat map showing the relative abundance of cell wall glycan epitopes released sequentially from the AIR fractions of non-induced and BL-induced suspension cells. Figure S3. Measurement of wall-bound phenolic acids in non-induced and BL-induced switchgrass suspension culture cells. Figure S4. Principal component analysis (PCA) of genes expressed in BL-induced cultures. Figure S5. The expression of CESA genes in non-induced and BL-induced cultures. Figure S6. Validation of cell wall-related gene expression in switchgrass suspension cultures by qRT-PCR. Figure S7. Overview of changes in transcript levels for genes involved in cell wall precursor synthesis in BL-induced cultures. Figure S8. Proposed route for S lignin biosynthesis in BL-induced suspension cells. [file 13068_2017_954_MOESM1_ESM.pdf]

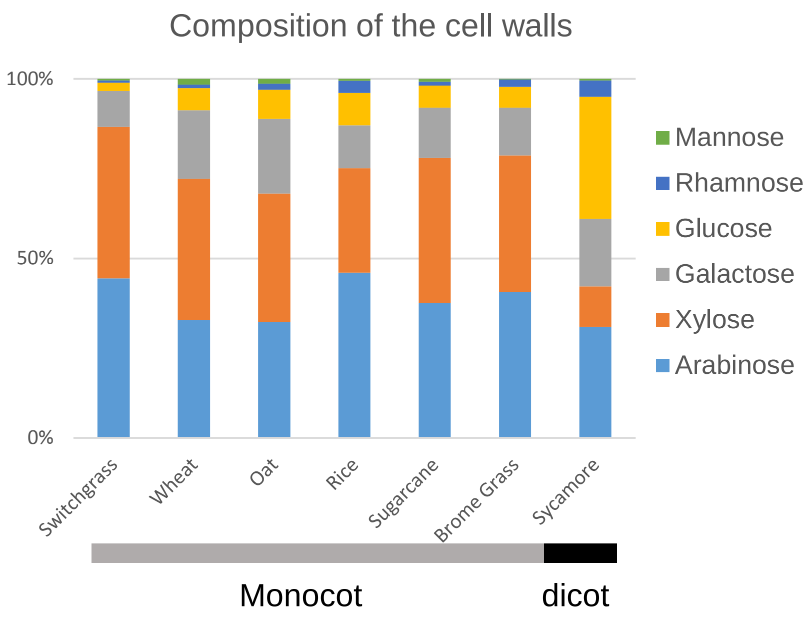

Figure S1. Comparison of the monosaccharide composition of switchgrass suspension cell walls with those of other plant cell suspensions. The data for cell culture from wheat, oat, rice, sugarcane, bromegrass and sycamore are from (Burke *et al*, 1974).

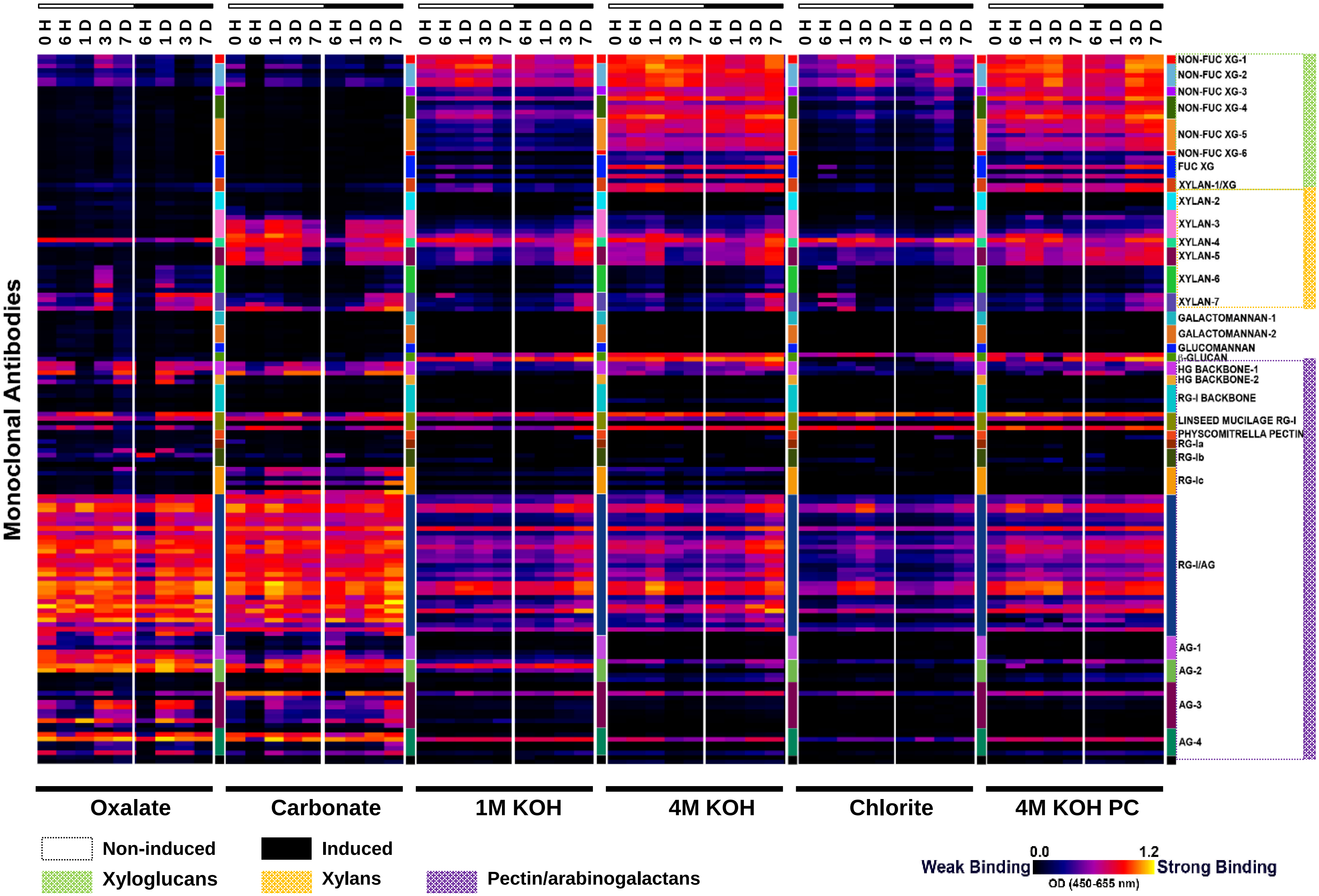

Figure S2. Glycome profiling heat map showing the relative abundance of cell wall glycan epitopes released sequentially from the AIR fractions of non-induced and BL-induced suspension cells.

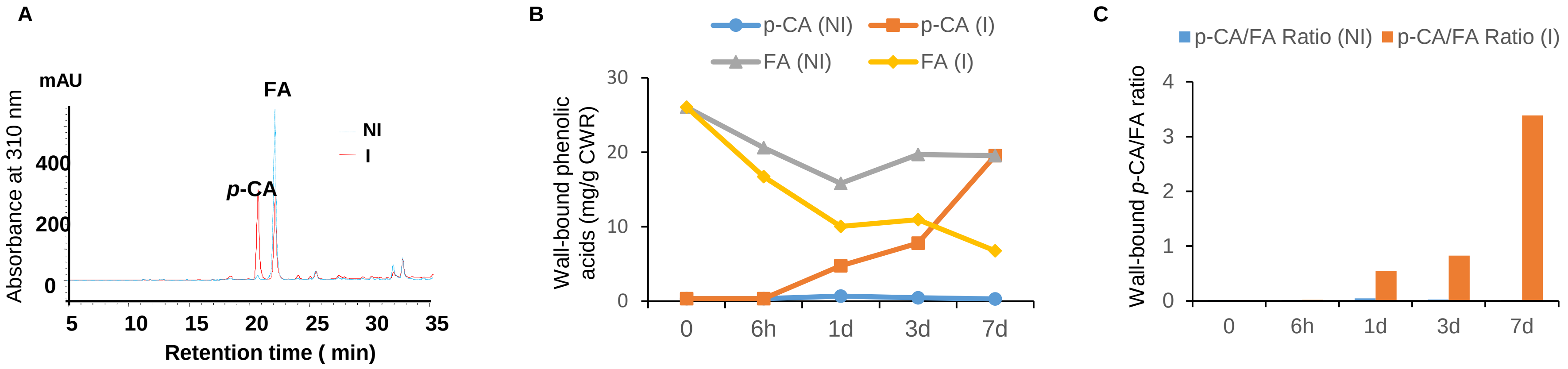

Figure S3. Measurement of wall-bound phenolic acids in non-induced and BL-induced switchgrass suspension cell culture.

A, HPLC chromatogram of ester-linked wall bound phenolics from induced (I) and non-induced (NI) cultures harvested at 3d. B, Levels of ester-linked wall-bound p-CA and FA in induced (I) and non-induced (NI) cells. C, Ester-linked wall-bound p-CA/FA ratio. p-CA, p-coumaric acid. FA, ferulic acid. The wavelength is p-CA, 310 nm and FA, 352 nm.

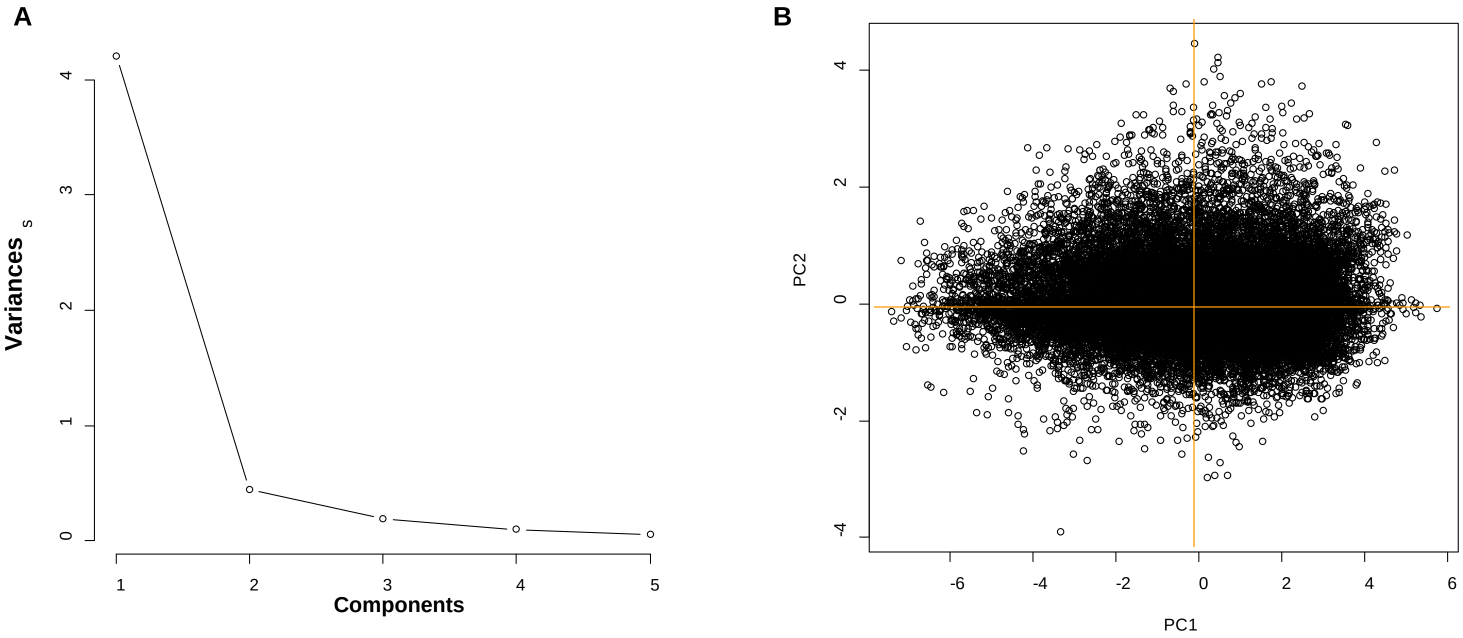

Figure S4. Principal component (PCA analysis) of genes expressed in BL-induced cultures.
A, Plot of variance of five primary components. B, Plot of dimensionally reduced expression data on the first and secondary primary components. The first component is the weighted average expression, and the second component is the changes in expression over time.

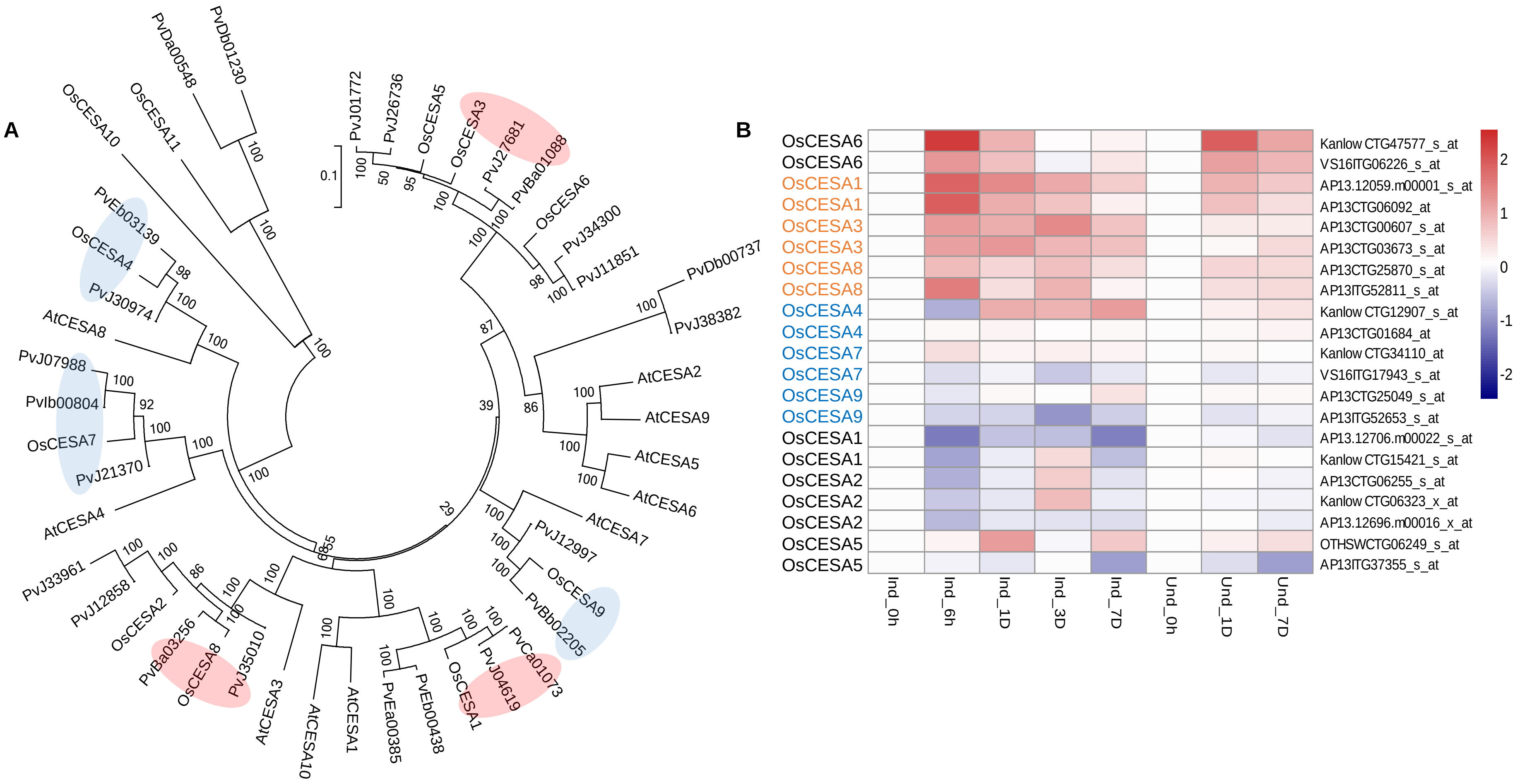

Figure S5. The expression of CESA genes in non-induced and BL-induced cultures.
A, Phylogenetic tree of CESA genes in switchgrass, rice and Arabidopsis. B, Expression of candidate CESA genes in switchgrass suspension cells.

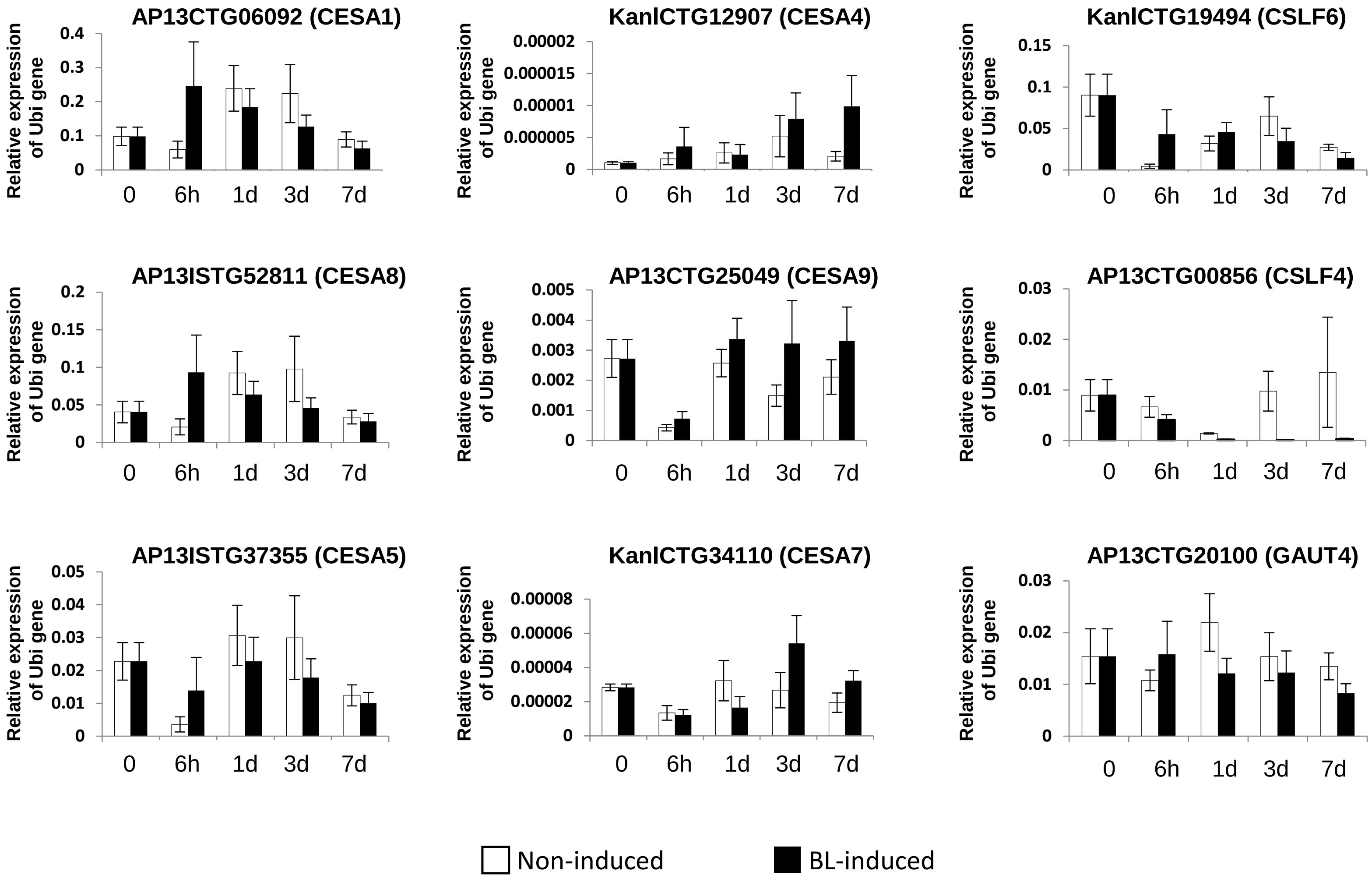

Figure S6. Validation of cell wall-related gene expression in switchgrass suspension cultures by qRT-PCR. Non-induced and induced samples are presented as open bars and closed bars, respectively. All data are means ± SE (n = 3).

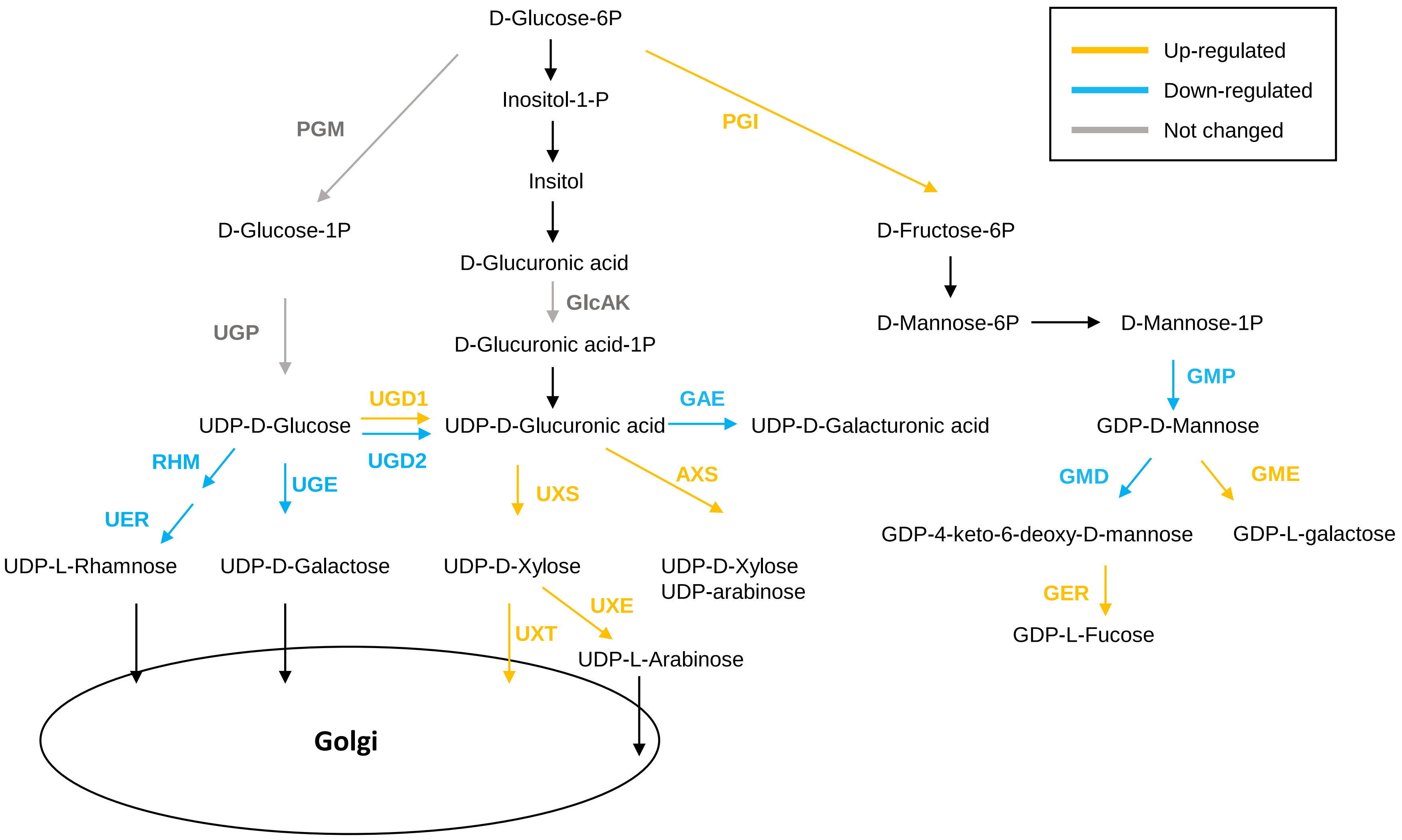

Figure S7. Overview of changes in transcript levels for genes involved in cell wall precursor synthesis in BL-induced cultures. Orange, blue and gray arrow indicate up-regulation, down-regulation and no significant change of gene expression after BL treatment in switchgrass suspension cells, respectively. PGM, phosphoglucomutase; PGI, glucose-6-phosphate isomerase; UGP, UDP-glucose pyrophosphorylase; GlcAK, glucuronokinase; UGD, UDP-glucose dehydrogenase; RHM, UDP-rhamnose synthase; UER, nucleotide-rhamnose synthase/epimerase-reductase; UGE, UDP-galactose-epimerase; UXS, UDP-xylose synthase; UXT, UDP-xylose transporter; UXE, UDP-xylose epimerase; AXS, UDP-apiose/xylose synthase; GAE, UDP-glucuronate-epimerase; GMP, GDP-mannose pyrophosphorylase; GMD, GDP-mannose-dehydratase; GME, GDP-mannose-epimerase; GER, GDP-4-keto-6-deoxymannose epimerase/reductase.

A

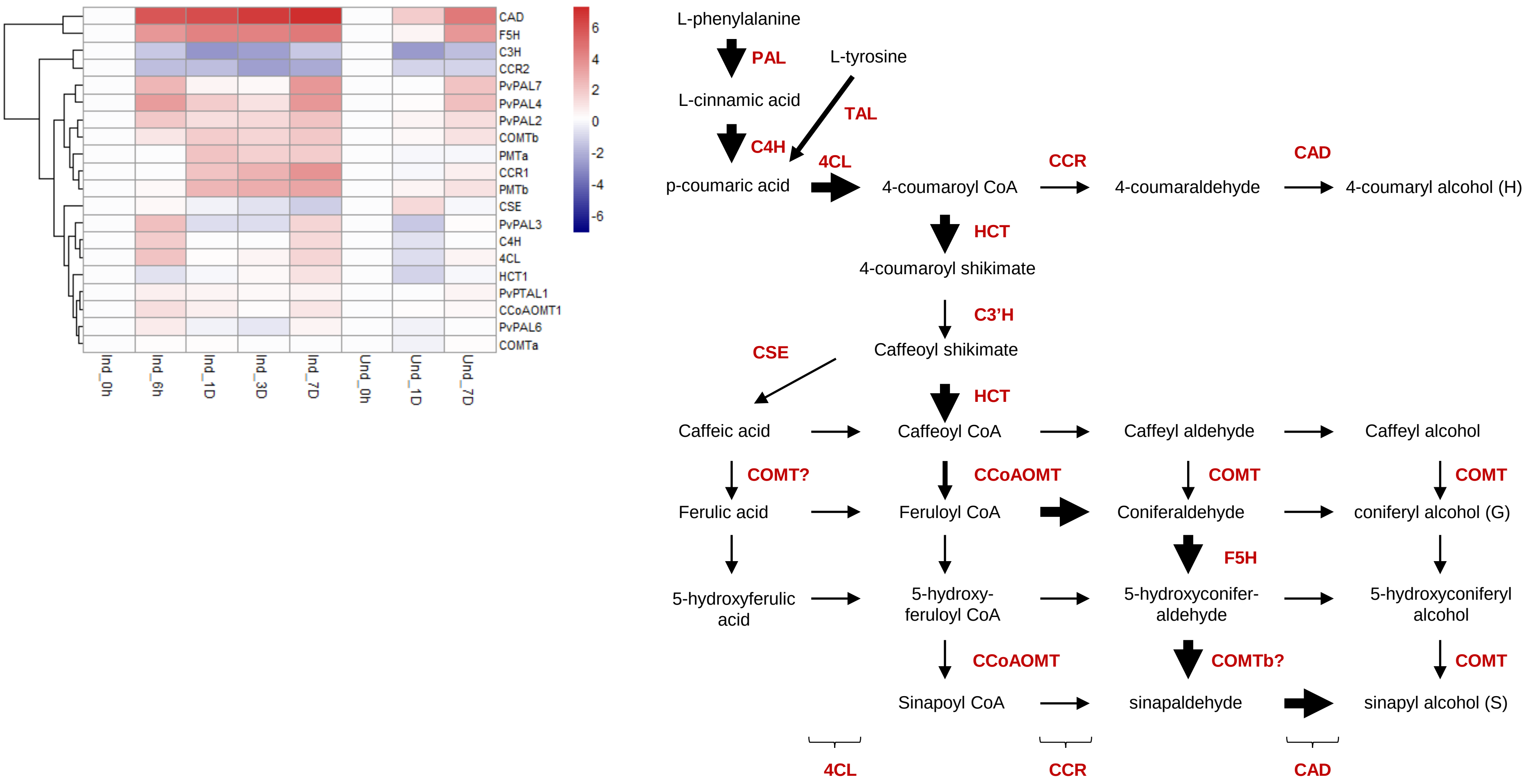

Figure S8. Proposed route for S lignin biosynthesis in BL-induced suspension cells.
A, heat map showing changes in expression of key lignin pathway genes. B, preferred pathway based on the data in A.
