# Supplementary material for: Dynamic changes in transcriptome and cell wall composition underlying brassinosteroid-mediated lignification of switchgrass suspension cells
Source: Biotechnol Biofuels. 2017 Nov 30;10:266. doi: 10.1186/s13068-017-0954-2 (PMC5707915; doi:10.1186/s13068-017-0954-2)
Supplement: Supplementary file 2 — Additional file 2: Materials and Methods. Supplementary information on pre-processing of the microarray dataset. [file 13068_2017_954_MOESM2_ESM.docx]

Additional Materials and Methods

**Functional classification of differentially expressed genes during BL-induced cell wall modification**

To obtain a genome-wide understanding of the transcriptional reprogramming associated with BL-induced changes in cell wall composition, microarray analyses were conducted on RNA extracted from cells harvested on 0 h, 6 h, 1 d, 3 d and 7 d for induced suspension cell cultures, as well as 1 d and 7 d for non-induced cultures [1]. The Affymetrix switchgrass cDNA microarray chip contains 122,973 probe sets corresponding to 110,208 expressed sequence tag (EST) sequences (PviUT database) [2]. After Robust Multiarray Average (RMA) normalization, we obtained the gene expression profiles with average value of 191 for each probe. We removed probes with maximum expression level less than 50 (defined as low expression genes) and 58,079 probes remained for subsequent analysis. Given that the existing annotation of the switchgrass microarray chip was generated in 2012 [2], but that a draft genome sequence (version 1.1) for switchgrass was released in 2015, we re-annotated the oligo probes on the chip through identifying their corresponding genes in the switchgrass reference genome v1.1 using BLAST.

To detect differentially expressed genes between the five time points following BL-treatment, we further filtered out gene sets with less than a 2-fold change in expression between samples. After filtering, 31,059 probe sets remained. As a preprocessing step to cluster and to visually summarize the features of the data, principal component analysis (PCA) was performed on the matrix containing the 31,059 rows of transcript level profiles and 5 columns of time points reflecting the growth stage of the induced suspension cultures. The majority (>80%) of variance was contributed by two variables (Fig. S4A); the first component represents the weighted average expression and the second represents the changes in expression over time [3]. The unimodal distribution of expression in the first and secondary principal components (Fig. S4B) suggests that the genes should be better classified by the method of neighbor determination of a gene [3].

**References**

1. Shen H, Mazarei M, Hisano H, Escamilla-Trevino L, Fu CX, Pu YQ, et al. A genomics approach to deciphering lignin biosynthesis in switchgrass*.* Plant Cell. 2013;25(11):4342-4361.

2. Zhang JY, Lee YC, Torres‐Jerez I, Wang M, Yin Y, Chou WC, et al. Development of an integrated transcript sequence database and a gene expression atlas for gene discovery and analysis in switchgrass (*Panicum virgatum* L.)*.* Plant J. 2013;74(1):160-173.

3. Raychaudhuri S, Stuart JM, and Altman RB. PRINCIPAL COMPONENTS ANALYSIS TO SUMMARIZE MICROARRAY EXPERIMENTS: APPLICATION TO SPORULATION TIME SERIES*.* Pacific Symposium on Biocomputing. Pacific Symposium on Biocomputing. 2000:455-466.
