# Supplementary material for: Dynamic changes in transcriptome and cell wall composition underlying brassinosteroid-mediated lignification of switchgrass suspension cells
Source: Biotechnol Biofuels. 2017 Nov 30;10:266. doi: 10.1186/s13068-017-0954-2 (PMC5707915; doi:10.1186/s13068-017-0954-2)
Supplement: Supplementary file 7 — Additional file 7: Results and Discussion. Supplementary information on genes involved in hormone signaling and metabolism, and biotic stress. [file 13068_2017_954_MOESM7_ESM.docx]

Additional Results and Discussion

**Genes involved in hormone signaling and metabolism**

To better understand how brassinosteroid signaling mediates cell wall biogenesis in suspension cells, we further searched for differentially regulated candidate genes involved in hormone transport, signaling and metabolism in our datasets (Additional file 5: Table S3). Several switchgrass genes encoding calcium transporters were up-regulated at 6 h in induced samples, whereas expression of the homolog of Arabidopsis ACA8 (autoinhibited Ca^2+^ -ATPase) was significantly down-regulated (Additional file 10: Table S7). ACA8 is thought to play a role in sucrose signaling [1]. We hypothesize that Ca^2+^ transport-mediated Ca^2+^ homeostasis might be a developmental cue in BL-induced suspension cultures.

A rapid increase in expression at 6 h and subsequent reduced expression of a homolog of Arabidopsis BAS1 (at2g26710) was observed in BL-induced cultures (Additional file 10: Table S7). BAS1 encodes a BR-inactivating enzyme [2]. Increased BAS1 expression upon BR perception is considered to be part of a feedback system that represses the activity of this hormone [3]. A long-term feedback loop is also observed in genes involved in BR biosynthesis. Expression of AP13CTG14609_s_at, a homolog of Arabidopsis BR6OX2 (At3g30180) that encodes a cytochrome P450 enzyme catalyzing the last reaction in the production of brassinolide [4], was down-regulated in response to BL. A similar response was found for a homolog of Arabidopsis SMT1 (at5g13710) that encodes an enzyme involved in controlling the level of cholesterol [4]. Expression of the BR6OX2 and SMT1 genes was not affected or only slightly reduced on days 1 and 7 in non-induced cultures. Homologs of the BR signaling receptors BES1 and BRI1 were expressed at lower level in induced than in non-induced cells at day 1. Together, these data suggest a feedback regulation in BL-treated suspension cells for reducing endogenous BL synthesis and decreasing BL signaling outputs.

Differential expression of genes involved in abscisic acid (ABA), salicylic acid (SA), jasmonic acid (JA) and gibberellic acid (GA) signaling and metabolism was also observed between induced and non-induced cells (Additional file 5: Table S3). For example, a homolog of GA2OX, encoding the enzyme for conversion of active GAs to inactive forms [5], was significantly downregulated in induced cells, and expressed at lower levels on days 1 and 7 than in non-induced cells (Additional file 10: Table S7). Expression of a homolog of SGT1, whose product catalyzes the formation of GA conjugates [6], was decreased in induced cells but increased in non-induced cells (Additional file 10: Table S7). Together, these data suggest pathway cross-talk between BR and other phytohormone signaling pathways in induced cells.

**Genes involved in biotic stress**

Considering the connection between BR signaling, lignin formation and plant immune responses [7, 8], we analyzed the expression profiles of biotic stress-related genes, many of which are up-regulated during the time course and preferentially expressed in BL-induced cells (Additional file 5: Table S3); such genes include homologs of PR1 and PR4, marker genes for salicylate-mediated biotic-stress responses [9], and genes homologous to NHL family members that have been identified as signaling mediators in response to pathogen attack in plants [10] (Additional file 8: Table S5). Other pathogenesis-related (PR) genes include those encoding thaumatin superfamily protein and proteins containing CC-NBS-LRR, CC-NBS and NBS-LRR type motifs (Additional file 8: Table S5). All the candidate NHL member and PR genes were significantly more highly expressed in induced than in non-induced cells on day 1 and/or day 7.

Lignin accumulation and secondary cell wall formation play key roles in both plant development and response to biotic stress. Under normal conditions, lignin deposition in fibers and vessels is accompanied with tracheary element development, which is important for water transport in plants. Under biotic stress, lignin serves as a non-degradable mechanical barrier to block pathogen invasion [11]. In suspension-cultured systems of Arabidopsis, Zinnia (*Zinnia elegans*) and banana embryonic cells [12-15], exogenously supplied BL initiates tracheary element differentiation with increased lignin content. However, in our switchgrass BL-induced suspension cultures, we detected increased levels of lignin without tracheary element formation. Moreover, we detected the elevated expression of many genes involved in sensing and resistance to pathogen attack (Additional file 5: Table S3 and Additional file 8: Table S5) which did not occur in non-induced cells. This suggests a role for BR in the activation of lignin biosynthesis as a defense response.

We detected a predominant accumulation of S lignin monomers in induced suspension cells when compared with the lignin in stem internodes (Fig 5). A similar abundance of S lignin in cell walls of suspension cultures is observed in *Ginkgo biloba* and maize [16, 17]. It is possible that the higher S:G ratio in suspension cells may be caused by the lack of mechanical stress in the cell cultures, although it is also often observed that the lignin formed during defense responses is predominantly composed of a single monomer type (e.g. all S lignin is formed in wheat leaves following infection *with Puccinia graminins* f.sp*. tritici* [18]).

**References**

1. Zhang J, Zhang X, Wang R, and Li W. The plasma membrane-localised Ca2+-ATPase ACA8 plays a role in sucrose signalling involved in early seedling development in Arabidopsis*.* Plant Cell Rep. 2014;33(5):755-766.

2. Bell EM, Lin W-c, Husbands AY, Yu L, Jaganatha V, Jablonska B, et al. Arabidopsis lateral organ boundaries negatively regulates brassinosteroid accumulation to limit growth in organ boundaries*.* Proc Natl Acad Sci USA. 2012;109(51):21146-21151.

3. Zhao Y, Qi Z, and Berkowitz GA. Teaching an Old Hormone New Tricks: Cytosolic Ca2+ elevation involvement in plant brassinosteroid signal transduction cascades*.* Plant Physiol. 2013;163(2):555-565.

4. Clouse SD. Brassinosteroids*.* The Arabidopsis Book / American Society of Plant Biologists. 2011;9:e0151.

5. Sun T-p. Gibberellin metabolism, perception and signaling pathways in Arabidopsis*.* The Arabidopsis Book / American Society of Plant Biologists. 2008;6:e0103.

6. Zhang K, Halitschke R, Yin C, Liu C-J, and Gan S-S. Salicylic acid 3-hydroxylase regulates Arabidopsis leaf longevity by mediating salicylic acid catabolism*.* Proc Natl Acad Sci USA 2013;110(36):14807-14812.

7. Dixon RA. Stress-induced phenylpropanoid metabolism*.* Plant Cell. 1995;7(7):1085-1097.

8. De Bruyne L, Höfte M, and De Vleesschauwer D. Connecting growth and defense: The emerging roles of brassinosteroids and gibberellins in plant innate immunity*.* Mol Plant. 2014;7(6):943-959.

9. van de Mortel JE, de Vos RCH, Dekkers E, Pineda A, Guillod L, Bouwmeester K, et al. Metabolic and transcriptomic changes induced in Arabidopsis by the Rhizobacterium *Pseudomonas fluorescens* SS101*.* Plant Physiol. 2012;160(4):2173-2188.

10. Bao Y, Song W-M, and Zhang H-X. Role of Arabidopsis NHL family in ABA and stress response*.* Plant Signaling & Behavior. 2016;11(5):e1180493.

11. Moura JCMS, Bonine CAV, Viana JDF, Dornelas MC, and Mazzafera P. Abiotic and biotic stresses and changes in the lignin content and composition in plants*.* J Integr Plant Biol. 2010;52(4):360-376.

12. Yamamoto R, Fujioka S, Demura T, Takatsuto S, Yoshida S, and Fukuda H. Brassinosteroid levels increase drastically prior to morphogenesis of tracheary elements. Plant Physiol. 2001;125(2):556-63.

13. Negi S, Tak H, and Ganapathi TR. In vitro xylem vessel elements formation from banana embryogenic cells and expression analysis of vessel development-related genes. Plant Biotechnol Rep. 2015;9(2):47-54.

14. Oda Y, Mimura T, and Hasezawa S. Regulation of secondary cell wall development by cortical microtubules during tracheary element differentiation in Arabidopsis cell suspensions. Plant Physiol. 2005;137(3):1027-1036.

15. Kubo M, Udagawa M, Nishikubo N, Horiguchi G, Yamaguchi M, Ito J, et al. Transcription switches for protoxylem and metaxylem vessel formation. Genes Dev. 2005;19(16):1855-1860.

16. Mélida H, Largo‐Gosens A, Novo‐Uzal E, et al. Ectopic lignification in primary cellulose‐deficient cell walls of maize cell suspension cultures. J. Integr. Plant Biol. 2015; 57, 357-372.

17. Uzal EN, Gómez Ros LV, Pomar F, Bernal MA, Paradela A, Albar JP, Ros Barceló A. The presence of sinapyl lignin in Ginkgo biloba cell cultures changes our views of the evolution of lignin biosynthesis. Physiol. Plant. 2009;135, 196-213.

18. Menden B, Kohlhoff M, Moerschbacher BM. Wheat cells accumulate a syringyl-rich lignin during the hypersensitive resistance response. Phytochemistry 2007; 68:513--20
