# Supplementary material for: Dynamic changes in transcriptome and cell wall composition underlying brassinosteroid-mediated lignification of switchgrass suspension cells
Source: Biotechnol Biofuels. 2017 Nov 30;10:266. doi: 10.1186/s13068-017-0954-2 (PMC5707915; doi:10.1186/s13068-017-0954-2)
Supplement: Supplementary file 8 — Additional file 8: Table S7. Expression of switchgrass genes involved in hormone signaling. [file 13068_2017_954_MOESM8_ESM.docx]

**Table S7.** Expression of switchgrass genes involved in hormone signaling.

| Gene information | | | Expression information | | | | | | | | | |
| --- | --- | --- | --- | --- | --- | --- | --- | --- | --- | --- | --- | --- |
|  |  |  |  |  | Induced | | | | Non-induced | | Compare | |
| Microarray Probe | PvGene | Clade/Gene | Cluster | 0 h | 6 h | 1 D | 3 D | 7 D | 1 D | 7 D | 1 D | 7 D |
| **Calcium transport** | | | | | | | | | | | | |
| AP13CTG00761_s_at | Pavir.J38100 | ACA3 | 1 | 885 | 2180 | 988 | 942 | 1206 | 1312 | 1226 |  |  |
| AP13CTG01165_s_at | Pavir.Ga00047 | CAX2 | 2 | 228 | 929 | 474 | 495 | 520 | 615 | 479 |  |  |
| AP13CTG16915_at | Pavir.Ca00103 | CAX11 | 2 | 41 | 263 | 78 | 97 | 63 | 117 | 64 | -1 | 0 |
| AP13ITG74187_at | Pavir.Ia01940 | CAX7 | 8 | 70 | 136 | 303 | 294 | 323 | 96 | 93 | 1 | 1 |
| KanlowCTG14078_s_at | Pavir.J12114 | ACA8 | 13 | 910 | 162 | 324 | 258 | 139 | 1244 | 558 | -1 | 0 |
| **BR synthesis** | | | | | | | | | | | | |
| AP13CTG14609_s_at | Pavir.Ib03913 | BR6OX2 | 13 | 3083 | 54 | 69 | 75 | 182 | 3742 | 3371 | -1 | -1 |
| AP13ITG51712_s_at | Pavir.J29978 | SMT1 | 13 | 3468 | 1541 | 1521 | 2684 | 913 | 2818 | 2401 | -1 | -1 |
| **BR signaling** | | | | | | | | | | | | |
| AP13ITG42436_s_at | Pavir.Db01623 | BAS1 | 1 | 195 | 760 | 106 | 66 | 78 | 1887 | 417 | -1 | 0 |
| AP13CTG23818RC_at | Pavir.Bb03208 | BES1 | 6 | 98 | 111 | 70 | 86 | 129 | 195 | 155 | -1 | 0 |
| AP13CTG15066_s_at | Pavir.Eb02593 | BRI1 | 14 | 3692 | 322 | 891 | 545 | 904 | 2976 | 1951 | -1 | 0 |
| **ABA metabolism** | | | | | | | | | | | | |
| KanlowCTG10190_s_at | Pavir.J00256 | AREB1 | 3 | 66 | 282 | 116 | 263 | 158 | 121 | 128 | 0 | 1 |
| **GA metabolism** | | | | | | | | | | | | |
| AP13ITG60008_at | Pavir.Eb03210 | GA2OX | 13 | 1184 | 132 | 68 | 69 | 75 | 751 | 330 | -1 | -1 |
| **SA metabolism** | | | | | | | | | | | | |
| OTHSWSLT33399_s_at | Pavir.Ba01318 | SGT1 | 5 | 20 | 51 | 18 | 6 | 6 | 402 | 103 | -1 | 0 |

Expression values at each data point represents the mean of three biological replicates. Cluster represents the expression groups of filtered genes in induced samples defined by self-organizing map (SOM) method (Figure 6A). Differential expression genes between induced and non-induced samples on 1 d and 7 d were identified by the linear model in LIMMA (Ritchie et al., 2015); -1 (represented by dark blue shade), significant lower expression in induced samples than in non-induced samples, 1 (represented by light orange shade), significant higher expression in induced samples than in non-induced samples, 0, no change between induced and non-induced samples.
