# Supplementary material for: Dynamic changes in transcriptome and cell wall composition underlying brassinosteroid-mediated lignification of switchgrass suspension cells
Source: Biotechnol Biofuels. 2017 Nov 30;10:266. doi: 10.1186/s13068-017-0954-2 (PMC5707915; doi:10.1186/s13068-017-0954-2)
Supplement: Supplementary file 9 — Additional file 9: Table S5. Expression of switchgrass genes involved in biotic stress responses. [file 13068_2017_954_MOESM9_ESM.docx]

**Table S5.** Expression of switchgrass genes involved in biotic-stress responses.

| Gene information | | | Expression information | | | | | | | | | |
| --- | --- | --- | --- | --- | --- | --- | --- | --- | --- | --- | --- | --- |
|  |  |  |  |  | Induced | | | | Non-induced | | Compare | |
| Microarray Probe | PvGene | Clade/Gene | Cluster | 0 h | 6 h | 1 D | 3 D | 7 D | 1 D | 7 D | 1 D | 7 D |
| **NHL family** | | | | | | | | | | | | |
| AP13ITG54121RC_at | Pavir.Fb00085 |  | 12 | 617 | 936 | 1764 | 2778 | 803 | 528 | 803 | 1 | 0 |
| AP13ITG60838_at | Pavir.J33033 |  | 12 | 18 | 35 | 283 | 1361 | 949 | 14 | 50 | 0 | 1 |
| **Pathogenesis-related thaumatin family** | | | | | | | | | | | | |
| AP13ITG57859-RC_at | Pavir.J24852 |  | 4 | 44 | 174 | 306 | 220 | 306 | 173 | 94 | 1 | 1 |
| KanlowCTG01069_s_at | Pavir.Ia03928 |  | 12 | 146 | 146 | 311 | 380 | 424 | 184 | 159 | 1 | 1 |
| AP13CTG27807_at | Pavir.Ia03929 |  | 12 | 63 | 58 | 368 | 333 | 240 | 62 | 102 | 1 | 0 |
| **Pathogenesis-related gene (marker gene)** | | | | | | | | | | | | |
| OTHSWSLT32350_s_at | Pavir.J38392 | PR1 | 11 | 36 | 28 | 39 | 36 | 145 | 70 | 34 | 0 | 1 |
| KanlowCTG46837_s_at | Pavir.J36772 | PR4 | 15 | 1010 | 329 | 305 | 2180 | 2382 | 233 | 160 | 0 | 1 |
| **Pathogenesis-recongize and resistance gene** | | | | | | | | | | | | |
| OTHSWCTG11465_at | Pavir.J12849 |  | 4 | 307 | 605 | 447 | 729 | 726 | 409 | 398 | 0 | 1 |
| AP13CTG01764_s_at | Pavir.J40356 |  | 4 | 163 | 317 | 459 | 484 | 495 | 165 | 270 | 1 | 1 |
| AP13CTG22951_s_at | Pavir.Fa01782 |  | 4 | 74 | 276 | 320 | 383 | 348 | 144 | 194 | 1 | 1 |
| AP13ITG39732_s_at | Pavir.J37164 |  | 2 | 116 | 271 | 149 | 162 | 299 | 175 | 170 | 0 | 1 |
| AP13ITG60036_at | Pavir.Ea01530 |  | 4 | 534 | 1652 | 1932 | 1833 | 2445 | 1717 | 1356 | 0 | 1 |
| **Disease resistance protein** | | | | | | | | | | | | |
| AP13ITG52744_at | Pavir.J10189 |  | 4 | 55 | 236 | 313 | 347 | 512 | 152 | 118 | 0 | 1 |
| AP13CTG35513_s_at | Pavir.J41099 | CC-NBS-LRR | 3 | 16 | 282 | 138 | 172 | 207 | 48 | 114 | 1 | 1 |
| AP13CTG02808_at | Pavir.Hb00191 | CC-NBS-LRR | 4 | 592 | 2210 | 2322 | 2684 | 2256 | 1509 | 1514 | 1 | 1 |
| AP13CTG15278_at | Pavir.Ea02999 | NBS-LRR | 4 | 282 | 402 | 463 | 505 | 600 | 307 | 416 | 1 | 1 |
| AP13CTG15479_s_at | Pavir.Fa00141 | NBS-LRR | 12 | 34 | 58 | 125 | 160 | 188 | 99 | 75 | 0 | 1 |
| AP13ITG53574_s_at | Pavir.Ha00262 | NBS-LRR | 3 | 885 | 3148 | 2261 | 2241 | 2718 | 1392 | 1625 | 1 | 1 |
| OTHSWCTG26966_s_at | Pavir.J39252 | NBS-LRR | 4 | 588 | 1218 | 1783 | 2539 | 2141 | 880 | 1088 | 0 | 1 |
| VS16ITG19460_at | Pavir.J04962 | NBS-LRR | 12 | 172 | 367 | 425 | 1342 | 904 | 201 | 182 | 0 | 1 |
| AP13CTG16437_s_at | Pavir.J12983 | CC-NBS | 12 | 184 | 345 | 632 | 1168 | 780 | 151 | 244 | 1 | 1 |
| AP13CTG46445_at | Pavir.J18888 | CC-NBS | 3 | 326 | 1160 | 686 | 781 | 937 | 214 | 822 | 1 | 0 |
| AP13ITG75844-RC_at | Pavir.Ha01469 | CC-NBS | 2 | 9 | 495 | 70 | 159 | 73 | 13 | 24 | 1 | 1 |
| AP13CTG18598_s_at | Pavir.Fa01855 | CC-NBS | 12 | 33 | 61 | 229 | 276 | 315 | 33 | 43 | 1 | 1 |

Expression values at each data point represents the mean of three biological replicates. Cluster represents the expression groups of filtered genes in induced samples defined by self-organizing map (SOM) method (Figure 6A). Differential expression genes between induced and non-induced samples on 1 d and 7 d were identified by the linear model in LIMMA (Ritchie et al., 2015); -1 (represented by dark blue shade), significant lower expression in induced samples than in non-induced samples, 1 (represented by light orange shade), significant higher expression in induced samples than in non-induced samples, 0, no change between induced and non-induced samples.
